# Distinct transcriptome signatures of *Helicobacter suis* and *Helicobacter heilmannii* strains upon adherence to human gastric epithelial cells

**DOI:** 10.1186/s13567-020-00786-w

**Published:** 2020-05-07

**Authors:** Helena Berlamont, Chloë De Witte, Eva Bauwens, Hannah Min Jou, Richard Ducatelle, Ellen De Meester, Yannick Gansemans, Dieter Deforce, Filip Van Nieuwerburgh, Freddy Haesebrouck, Annemieke Smet

**Affiliations:** 1 Department of Pathology, Bacteriology and Avian Diseases, Faculty of Veterinary Medicine, Ghent University, Merelbeke, Belgium; 2 Laboratory of Pharmaceutical Biotechnology, Faculty of Pharmaceutical Sciences, Ghent University, Ghent, Belgium; 3 Translational Research in Immunology and Inflammation, Laboratory of Experimental Medicine and Pediatrics, Faculty of Medicine and Health Sciences, Antwerp University, 2610 Antwerp, Belgium

## Abstract

The porcine *Helicobacter suis* and canine-feline *H. heilmannii* are gastric *Helicobacter* species with zoonotic potential. However, little is known about the pathogenesis of human infections with these *Helicobacter* species. To gain more insight into the interactions of both zoonotic *Helicobacter* species with human gastric epithelial cells, we investigated bacterial genes that are differentially expressed in a *H. suis* and *H. heilmannii* strain after adhesion to the human gastric epithelial cell line MKN7. In vitro *Helicobacter*-MKN7 binding assays were performed to obtain bacterial RNA for sequencing analysis. *H. suis* and *H. heilmannii* bacteria attached to the gastric epithelial cells (i.e. cases) as well as unbound bacteria (i.e. controls) were isolated, after which prokaryotic RNA was purified and sequenced. Differentially expressed genes were identified using the DESeq2 package and SARTools pipeline in R. A list of 134 (83 up-regulated and 51 down-regulated) and 143 (60 up-regulated and 83 down-regulated) differentially expressed genes (p_adj_ ≤ 0.01; fold change ≥ 2) were identified for the adherent *H. suis* and *H. heilmannii* strains, respectively. According to BLASTp analyses, only 2 genes were commonly up-regulated and 4 genes commonly down-regulated in both pathogens. Differentially expressed genes of the *H. suis* and *H. heilmannii* strains belonged to multiple functional classes, indicating that adhesion of both strains to human gastric epithelial cells evokes pleiotropic adaptive responses. Our results suggest that distinct pathways are involved in human gastric colonization of *H. suis* and *H. heilmannii*. Further research is needed to elucidate the clinical significance of these findings.

## Introduction

*Helicobacter* (*H*.) *pylori* is the best studied gastric *Helicobacter* species naturally colonizing more than half of the world’s human population. It is responsible for a wide range of gastric pathologies, including cancer [1, 2]. Other spiral-shaped non-*H. pylori Helicobacter* species (NHPH) have been demonstrated to colonize the human gastric mucosa as well. Their prevalence in humans ranges from 0.1 to 6.2% [3–5], with a higher density in Asia compared to Europe [5–7]. These NHPHs normally colonize the stomach of animals, but some have zoonotic potential. Particularly porcine *H. suis* strains and *H. heilmannii* strains, which are mainly associated with cats and dogs, have been described in human patients with gastritis, gastric or duodenal ulcers or low grade mucosa-associated lymphoid tissue (MALT) lymphoma [5, 8, 9]. Transmission from animals to humans may occur through direct or indirect contact with infected animals, and, in case of *H. suis*, also via consumption of raw or undercooked pork [8, 10].

Adherence to the gastric mucosa is the initial step in gastric colonization and pathogenesis of *Helicobacter* spp. infections [11, 12]. To identify which bacterial genes are involved in gastric colonization, gene expression profiles of *Helicobacter* bacteria attached to gastric epithelial cells need to be compared with those of unbound bacteria. Cell culture experiments may provide initial and valuable information concerning specific host-regulated genes that could subsequently be verified in vivo [13].

So far, transcriptome profiles of *H. suis and H. heilmannii* upon adherence to the human gastric epithelium have not been described. Previous studies mainly focused on *H. pylori*-induced changes in host cell gene expression and potential strain differences using the human gastric adenocarcinoma AGS cell line [13–18]. This cell line, however, does not form an organized cell layer with functional tight junctions [19]. In this study, we used the human gastric epithelial MKN7 cell line as it resembles more the gastric mucosa by the formation of a contiguous polarized monolayer displaying mucin expression [19, 20]. To gain more insights into the pathogenesis of animal-associated *Helicobacter* infections in humans, we compared the expression of *H. suis* and *H. heilmannii* genes upon binding to human gastric epithelial MKN7 cells to unbound *H. suis* and *H. heilmannii*. RNA sequencing approaches were used as they provide a more precise, annotation-independent measurement of transcript levels, compared to other transcriptome profiling methods such as hybridization-based methods [21, 22]. Genes that are significantly up-regulated upon binding to gastric epithelial cells might play a role in gastric pathogenesis and in the ability of *Helicobacter* to have effects beyond the stomach as well.

## Materials and methods

### Bacterial strains and growth conditions

The porcine *H. suis* type strain HS1 (LMG 23995^T^; DSM 19735^T^) and the feline *H. heilmannii* type strain ASB1 (LMG 26292^T^, DSM 24751^T^) were used in this study [23, 24]. The bacteria were grown under microaerobic conditions (85% N_2_, 10% CO_2_, 5% O_2_) at 37 °C on biphasic *Brucella* agar plates (Becton–Dickinson, Erembodegem, Belgium) supplemented with 20% fetal calf serum (HyClone, Logan, UT, USA), 5 mg amphotericin B/I (Fungizone, Bristol-Myers Squibb, Epernon, France), *Campylobacter* selective supplement (Skirrow, Oxoid, Basingstoke, UK; containing 10 mg/L vancomycin, 5 mg/L trimethoprim lactate and 2500 U/L Polymyxin B) and Vitox supplement (Oxoid). *Brucella* broth (Oxoid) was added on top of the agar to obtain biphasic culture conditions. The pH of both agar and broth was adjusted to 5 by adding HCl to a final concentration of 0.05%. After incubation, the bacteria were harvested and the concentration was determined using an improved Neubauer counting chamber (Sigma-Aldrich, Saint Louis, Missouri, USA).

### *H. suis* and *H. heilmannii* fluorescence-based adherence assay

A quantitative fluorescence-based adherence assay was performed to gain insights into the binding capacity of the *H. suis* and *H. heilmannii* strains to the human gastric epithelial cell line MKN7 (Riken Cell Bank, Tsukuba, Japan). MKN7 cells were seeded at a concentration of 1 × 10^6^ cells/mL in 200 µL antibiotic-free cell medium (89% RPMI medium 1640 supplemented with 1 mM 1-glutamine (Invitrogen, Waltham, MA, USA) and 10% fetal calf serum (FCS; HyClone, Logan, Utah, USA) in 96-well plates (Greiner-Bio One, Vilvoorde, Belgium) and incubated overnight at 37 °C. The HS1 and ASB1 strains were fluorescently labeled with fluorescein isothiocyanate isomer 1 (FITC, λ_ex_ 492 nm, λ_em_ 518 nm; Sigma-Aldrich). Briefly, bacteria were cultivated as described above and harvested at a concentration of 3.3 × 10^8^ viable bacteria/mL *Brucella* broth. Next, bacteria were washed 3 times in PBS-Tween 0.05% (2000 *g* for 5 min) and pellets were resuspended in 0.1 M carbonate and 0.15 M NaCl buffer (pH 9). Subsequently, 10 µL FITC (10 mg/mL dimethyl sulfoxide (DMSO)) was added to 1 mL bacterial suspension. After incubation for 30 min in the dark, FITC-labeled bacteria were washed 3 times in blocking buffer (1% bovine serum albumin (BSA) in PBS-Tween 0.05%). Viability of FITC-labeled bacteria was ascertained by checking their motility using light microscopy. After removing antibiotic-free cell medium from the 96-well plates, 150 µL of FITC-labeled bacterial suspension was added to the cells (5 replicates per strain), followed by incubation for 1 h at 37 °C under microaerobic conditions. Subsequently, cells were washed twice with HBSS + (Hank’s Balanced Salt Solution, Thermo Fisher Scientific, Waltham, Massachusetts, USA) and the emission of fluorescent light at λ = 527 nm was measured with a fluorimeter (Fluoroskan Ascent^TM^ FL Microplate Fluorometer and Luminometer, Thermo Scientific, Erembodegem-Aalst, Belgium). The adherence assay was performed immediately after fluorescent labeling of the *Helicobacter* strains in order to minimize the possible loss in viability of the labeled helicobacters over time. To correct for possible background signals, wells without cells (bacterial suspension only) and wells without bacterial suspension (MKN7 cells only) were included as controls. Additionally, the relative levels of FITC labeling of the HS1 and ASB1 strain were analyzed by flow cytometry (FCM) on a CytoFLEX flow cytometer (Beckman Coulter, Indiana, United States) and the mean fluorescence intensity of each labeled strain was used as a correction factor for differential FITC labeling of the strains. The ratio of the mean fluorescence intensity measured by the fluorimeter (indicating bacterial adhesion) to the mean fluorescence intensity measured by FCM (indicating the relative FITC labeling per strain) was calculated for each *Helicobacter* strain.

### RNA isolation of bound and unbound *Helicobacter* bacteria

To obtain RNA from *H. suis* and *H. heilmannii* strains incubated with the gastric cell line, MKN7 cells were seeded at a concentration of 1 × 10^6^ cells/mL in 2 mL antibiotic-free cell medium in 6-well plates (Greiner-Bio One, Vilvoorde, Belgium) and incubated for 24 h at 37 °C and 5% CO_2_ until a confluent monolayer was formed. Subsequently, 2 mL containing 6.6 × 10^8^ viable *H. suis* or *H. heilmannii* bacteria were added to the wells and incubated for 8 h under microaerobic conditions. After checking the MKN7 confluency, wells were washed 3 times with HBSS + after which 1 mL of 1% trypsin solution consisting of 88% trypsin diluent (8 g NaCl, 0.2 g KCl, 0.12 g KH_2_PO_4_, 0.91 g Na_2_HPO_4_, and 4 mL 0.5% phenol red solution/1000 mL aqua dest), 10% trypsin stock solution (Invitrogen), and 2% EDTA (2 g/100 mL trypsin diluent) was added to the wells followed by an incubation of 10 min. Next, 1 mL of cell medium without antibiotics was added and the content of the wells was collected, centrifugated (3 min, 1300 *g*, 4 °C) after which the pellet was stored at −70 °C until RNA extraction. For the RNA isolation of *H. suis* and *H. heilmannii* in the absence of gastric epithelial cells, wells without MKN7 cells were included and 2 mL containing 6.6 × 10^8^ viable *H. suis* or *H. heilmannii* bacteria were added to the wells. After 8 h of incubation under microaerobic conditions, bacterial suspension was collected, centrifugated (3 min, 1300 *g*, 4 °C), and the pellet was stored at −70 °C until RNA extraction.

From all collected samples, RNA was then extracted using the RNeasy mini kit (Qiagen, Venlo, the Netherlands) according to the manufacturer’s instructions and RNA-yield was measured using Nanodrop equipment (Nanodrop ND-1000, Fisher Scientifics, Hampton, New Hampshire, USA).

Concentration and quality of the total extracted RNA was checked by using the Quant-it ribogreen RNA assay (Life Technologies, Carlsbad, California, USA) and the RNA 6000 nano chip (Agilent Technologies, Santa Clara, California, USA), respectively.

### RNA sequencing and downstream bioinformatics approaches

The NEBNext^®^ Ultra^TM^ RNA Library Prep Kit for Illumina^®^ (New England Biolabs, Ipswich, Massachusetts, USA) with rRNA depletion was used for directional total RNA library preparation. Sequencing was performed on the NextSeq 500 High output system (Illumina, San Diego, California, USA).

The obtained raw sequencing reads were trimmed for sequencing adaptors, low quality and ambiguous bases using cutadapt (v1.11) [25]. Initial and final read quality was checked with FastQC (v0.11.5). To remove contaminating reads mapping to human DNA, the trimmed reads were aligned on the human reference genome (GRCh38) using the STAR aligner (v2.5.3) [26] with recovery of unmapped reads.

Reads not mapping on the human genome were mapped against the *H. suis* HS1 (NCBI accession: NZ_ADGY00000000) and the *H. heilmannii* ASB1.4 (EMBL accession: HE984298) reference genomes using the Bowtie2 aligner (v2.2.5) [27]. Identification of mRNA features and counting was done with featureCounts from the Subread package (v1.5.3) [28] using the *Helicobacter* mappings and reference genome annotations as inputs.

Differential gene expression between case (i.e. *H. suis* and *H. heilmannii* attached to MKN7 cells) and control (i.e. unbound bacteria) samples was performed in the statistical programming environment R [29], using the DESeq2 package [30] and the SARTools pipeline [31]. Differentially expressed features having a p_adj_ ≤ 0.01 and a fold change of at least 2 were identified. A volcano and PCA plot were generated to visualize the differentially expressed genes.

A reciprocal BLAST was performed at the protein level. BLAStp with default parameters was used to identify homologs and to compare differentially expressed genes of *H. suis* and *H. heilmannii*. Homology was assumed when BLASTp hits of *H. suis* and *H. heilmannii* protein sequences had an e-value below 10 to avoid missing homologs. However, as this criterium alone is not very stringent, we also considered other homology quality metrics like (number of gaps, % identity and % positive match to assess the quality of the match.

## Results

### Binding capacity of *H. suis* and *H. heilmannii* to MKN7 cells

**Figure 1:**
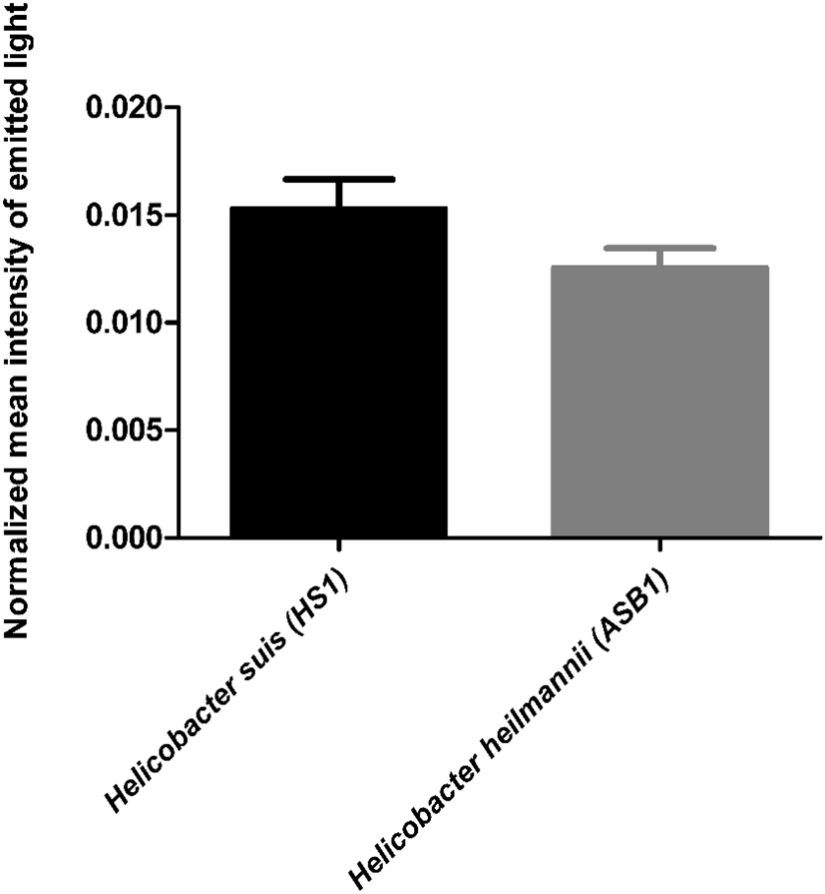
**Fluorescence-based adherence assay of*H. suis*and*H. heilmannii*isolates to gastric epithelial MKN7 cells.** The binding of FITC-labeled *H. suis* (HS1) and *H. heilmannii* (ASB1) isolates to gastric epithelial MKN7 cells was quantified by measuring the emission of fluorescent light at λ = 527 nm. The relative levels of FITC labeling of HS1 and ASB1 were analyzed by flow cytometry to correct for differential labeling of both *Helicobacter* strains. Therefore, data are presented as the mean intensity of the emitted light normalized to the relative level of FITC-labeling of *H. suis* and *H. heilmannii* strains.

A quantitative fluorescence-based adherence assay was performed to compare binding capacity of *H. suis* HS1 and *H. heilmannii* ASB1 to human gastric MKN7 cells. As shown in Figure 1, the mean intensity of fluorescent light emitted after binding of FITC-labeled *H. suis* and *H. heilmannii* bacteria to MKN7 cells was not significantly different. Given that the intensity of emitted light is proportional with the quantity of cell-bound bacteria, this experiment shows a similar binding capacity of *H. suis* and *H. heilmannii* to gastric epithelial cells.

### Differential gene expression analysis of *H. suis* adhering to MKN7 cells

When mapping the obtained reads against the human GRCh38 genome (EMBL accession: GCA_000001405.28), we observed that 95–97% of the reads from the *H. suis*-MKN7 coculture samples (i.e. cases) mapped against the human genome, compared to 0.4% of the reads from unbound *H. suis* samples (i.e. controls). Conversely, only 3–4% of the reads from *H. suis*-MKN7 coculture samples (i.e. cases) mapped against the *H. suis* HS1, compared to 95–96% of the reads from the unbound *H. suis* (i.e. controls).

**Figure 2:**
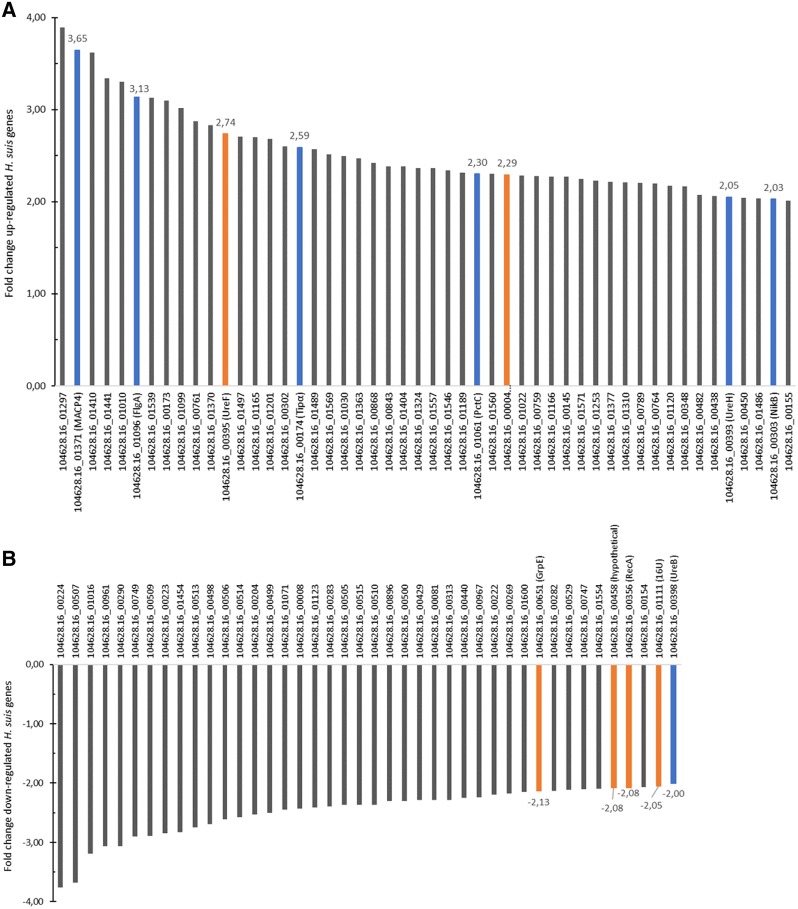

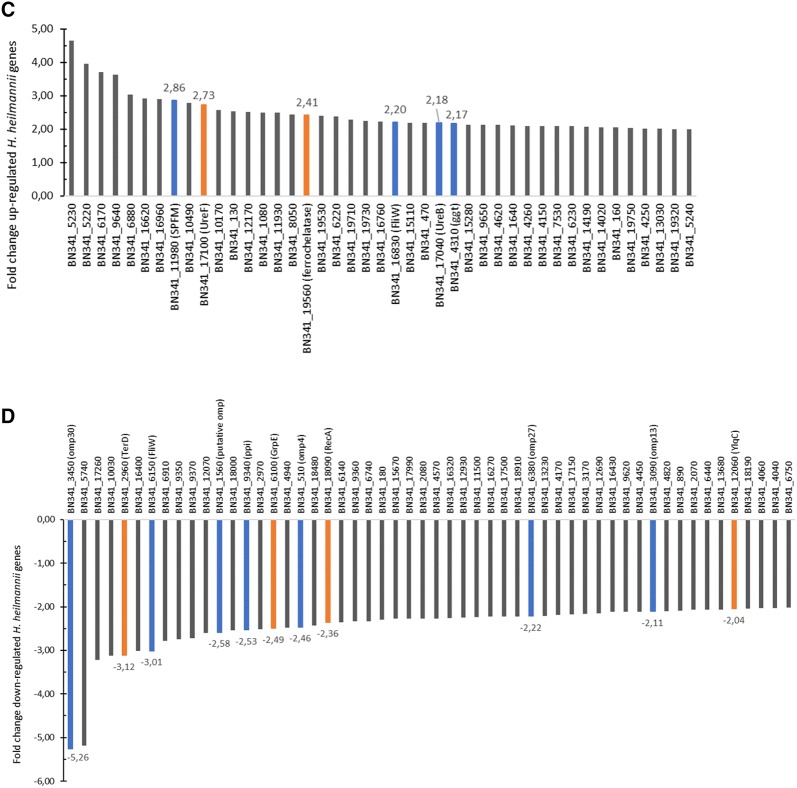
**Fold change data of up- and down-regulated genes of MKN7-bound*H. suis*and*H. heilmannii*(i.e. cases) compared to unbound*H. suis*and*H. heilmannii*(i.e. controls). A** Up-regulated *H. suis* genes. **B** Down-regulated *H. suis* genes. **C** Up-regulated *H. heilmannii* genes. **D** Down-regulated *H. heilmannii* genes. **A**, **B** Fold change data of up- (**A**) and down-regulated (**B**) *H. suis* genes (hypothetical protein genes excluded, except for *H. suis* 104628.16_00458) (with p_adj_ ≤ 0.01; fold change ≥ 2). **A** The up-regulation of 50 *H. suis* genes (33 hypothetical protein genes excluded) ranged from 2.01- to 3.89-fold. **B** The down-regulation of 42 *H. suis* genes (9 hypothetical protein genes excluded) ranged from 2.0- to 3.76-fold. **C**, **D** Fold change data of up- (**C**) and down-regulated (**D**) *H. heilmannii* genes (hypothetical protein genes excluded) (with p_adj_ ≤ 0.01; fold change ≥ 2). **C** The up-regulation of 43 *H. heilmannii* genes (17 hypothetical protein genes excluded) ranged from 2- to 4.64-fold. **D** The down-regulation of 54 genes (29 hypothetical protein genes excluded) ranged from 2.02- to 5.26-fold. Common up- or down-regulated genes in both MKN7-bound *H. suis* and *H. heilmannii* according to the reciprocal BLASTp analysis are displayed in orange. Interesting genes probably associated with virulence and colonization capacity are displayed in blue. 16U: general stress protein 16U gene; *FlgA*: flagellar basal body P-ring biosynthesis protein gene; *FliW*: flagellar assembly factor FliW gene*; ggt*: gamma-glutamyl transpeptidase gene; *GrpE:* heat shock protein GrpE gene; MACP4: methyl-accepting chemotaxis protein 4 gene; *NikB*: nickel transport system permease protein gene; *omp*: outer membrane protein gene; *PctC*: methyl-accepting chemotaxis protein PctC gene*; ppi*: peptidyl-prolyl *cis,trans*-isomerase gene; *RecA*: recombinase A gene; SPFM: secreted protein involved in flagellar motility gene; *TerD*: tellurium resistance gene; *Tipα*: tumor necrosis factor-α inducing protein gene; *UreB*: urease subunit beta gene; *UreF*: urease accessory protein UreF gene; *UreH*: urease accessory protein UreH gene; *YlqC*: KH domain RNA binding protein gene.

Using the SARTools wrapper for DESeq2, a list of 539 differentially expressed genes (p_adj_ ≤ 0.05) between case (i.e. bound bacteria) and control (i.e. unbound bacteria) groups was generated for *H. suis*. After refining the results to those having a p_adj_ ≤ 0.01 and a fold change of at least 2, a list of 134 (Additional files 1, 2) differentially expressed genes between case and control groups was obtained. 83 genes were up-regulated (Additional file 1) and 51 were down-regulated (Additional file 2) upon binding to MKN7 cells compared to unbound bacteria. Figures 2A and B display the fold change data of the up- and down-regulated genes (hypothetical protein genes excluded), respectively, in cases compared to controls.

### Differential gene expression analysis of *H. heilmannii* adhering to MKN7 cells

When mapping the obtained reads against the human GRCh38 genome (EMBL accession: GCA_000001405.28), 86-90% of the reads from the *H. heilmannii*-MKN7 coculture samples (i.e. cases) mapped against the human genome, compared to 0.1% of the reads from unbound *H. heilmannii* samples (i.e. controls). Conversely, only 4–9% of the reads from the *H. heilmannii*-MKN7 coculture samples (i.e. cases) mapped against the *H. heilmannii* ASB1.4 genome, compared to 96–97% of the reads from the unbound *H. heilmannii* samples (i.e. controls).

With the SARTools wrapper for DESeq2, a list of 773 differentially expressed genes (p_adj_ ≤ 0.05) between case (i.e. bound bacteria) and control (i.e. unbound bacteria) groups was generated for *H. heilmannii*. After refining the results to those having a p_adj_ ≤ 0.01 and a fold change of at least 2, a list of 143 (Additional files 3, 4) differentially expressed genes between case and control groups was obtained. 60 genes were up-regulated (Additional file 3) and 83 were down-regulated (Additional file 4) when bound to MKN7 cells compared to unbound bacteria. Figures 2C and D display the fold change data of the up- and down-regulated genes (hypothetical protein genes excluded), respectively, in cases compared to controls.

### Graphical display of RNA sequencing results for *H. suis* and *H. heilmannii*

The volcano plots of the comparisons (case versus control) for both *H. suis* and *H. heilmannii* are displayed in Additional file 5. The volcano plots represent the log of the adjusted p-value as a function of the log ratio of differential expression, enabling a quick visualization of those data points that display large magnitude changes that are also statistically significant. Significantly differentially expressed features (p_adj_ ≤ 0.01, fold change ≥ 2) are represented by red dots. Dots toward the top of the volvano plot represent highly significantly differentially expressed genes, and dots at either the left- or right-hand side of the volcano plot represent values that display large magnitude fold changes.

To check whether the main variability within each experiment came from biological differences between case and control samples, the first principal components (PC1 and PC2) of the PCA (Principal Component Analysis) were checked (Additional file 6). The first principal component (PC1) separates samples from different biological conditions. In both the PCA-plot for *H. suis*. (Additional file 6A) and the PCA-plot for *H. heilmannii* (Additional file 6B), the sample groups (i.e. case and control) were well separated, indicating that biological variability was the main source of variance in the data.

### Comparative analysis of the differentially expressed *H. suis* genes with the *H. heilmannii* genome

**Figure 3:**
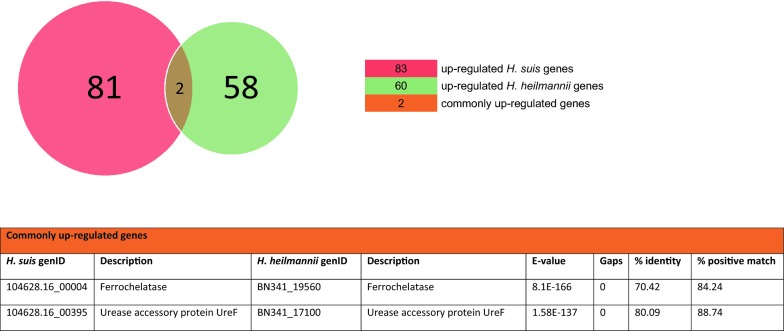
**Commonly up-regulated*H. suis*and*H. heilmannii*genes according to reciprocal BLASTp.**

Reciprocal BLASTp analysis of the differentially expressed *H. suis* genes with the *H. heilmannii* genome showed that 11 out of 83 up-regulated *H. suis* genes did not have homologs (i.e. BLASTp hits of *H. suis* and *H. heilmannii* protein sequences having e-values below 10) present in the *H. heilmannii* genome (Additional file 7). The 11 *H. suis* genes with no *H. heilmannii* homologs were mainly genes encoding unknown function, with the exception of one gene involved in oxidation–reduction (*YdgJ*) and one in DNA modification (Additional file 7). Seventy-two out of the 83 up-regulated *H. suis* genes had homologs present in the *H. heilmannii* genome (Additional file 7). Only 2 commonly up-regulated genes (i.e. the urease accessory protein gene (*UreF*) and ferrochelatase gene (displayed in orange in Figures 2A, C, and in Figures 3 and 5) among both species were identified (Additional files 1 and 3). One up-regulated *H. suis* gene (hypothetical protein gene) had a down-regulated *H. heilmannii* homolog (Figure 5).

**Figure 4:**
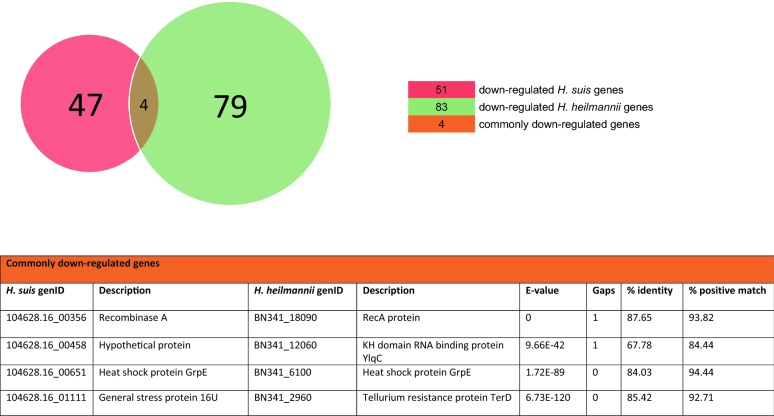
**Commonly down-regulated*H. suis*and*H. heilmannii*genes according to reciprocal BLASTp.**

**Figure 5:**
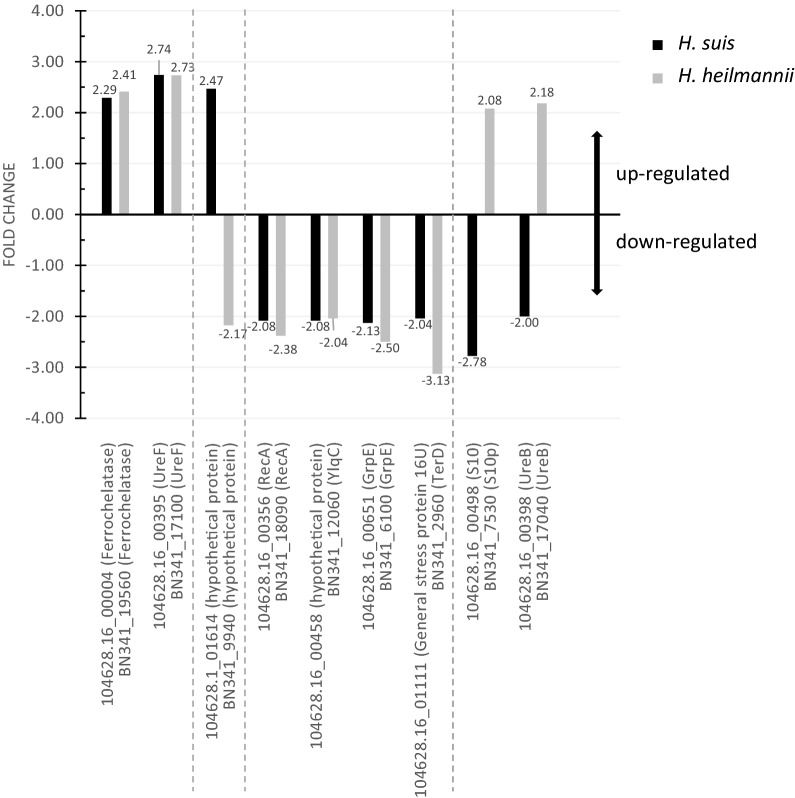
**Comparative analysis between the*H. suis*and*H. heilmannii*differentially expressed genes.**

Furthermore, 6 out of 51 down-regulated *H. suis* genes did not have homologs present in the *H. heilmannii* genome (Additional file 8). Although the function of these 6 genes is mostly unknown, 1 gene (i.e. 60 kDa chaperonin 1) is likely to be involved in protein folding (Additional file 8). Forty-five out of 51 down-regulated *H. suis* genes had *H. heilmannii* homologs (Additional file 8). Four genes (i.e. recombinase A gene (*RecA*), tellurium resistance gene (*TerD*) of *H. heilmannii* with its *H. suis* homolog general stress protein 16U gene, KH domain RNA binding protein gene (*YlqC*) of *H. heilmannii* with its *H. suis* homolog hypothetical protein gene, and heat shock protein gene (*GrpE*)) were commonly down-regulated in *H. suis* and *H. heilmannii* and are shown in orange in Figures 2B and D, and in Figures 4 and 5 (Additional files 2 and 4). Two down-regulated *H. suis* genes (i.e. 30S ribosomal protein S10 gene and *UreB*) had an up-regulated *H. heilmannii* homolog (Figure 5).

### Comparative analysis of the differentially expressed *H. heilmannii* genes with the *H. suis* genome

Reciprocal BLAST analysis of the differentially expressed *H. heilmannii* genes with the *H. suis* genome showed that 13 out of 60 up-regulated *H. heilmannii* genes did not have homologs present in the *H. suis* genome (Additional file 9). The function of most *H. heimannii* genes not having homologs in the *H. suis* genome is currently unknown, and only 2 genes involved in translation (*S16e*, *L13Ae*) were identified (Additional file 9). Of these 60 up-regulated *H. heilmannii* genes, 47 genes had homologs present in the *H. suis* genome (Additional file 9). Apart from the 2 earlier mentioned commonly up-regulated genes among both *Helicobacter* species, 2 up-regulated *H. heilmannii* genes (i.e. SSU ribosomal protein S10p (S20e) gene and *UreB*) had a down-regulated *H. suis* homolog (Figure 5).

Finally, 9 of the 83 down-regulated *H. heilmannii* genes did not have homologs present in the *H. suis* genome (Additional file 10) and encoded hypothetical proteins, except for one gene which is involved in metal ion transport (i.e. Mn^2+^/Fe^2+^ transporter gene). Seventy-four out of 83 down-regulated *H. heilmannii* genes had *H. suis* homologs, with, as previously mentioned, 4 genes commonly down-regulated among both *Helicobacter* species (Figures 4, 5 and orange in Figure 2).

### Comparative analysis between differentially expressed *H. suis* and *H. heilmannii* genes according to gene function

**Figure 6:**
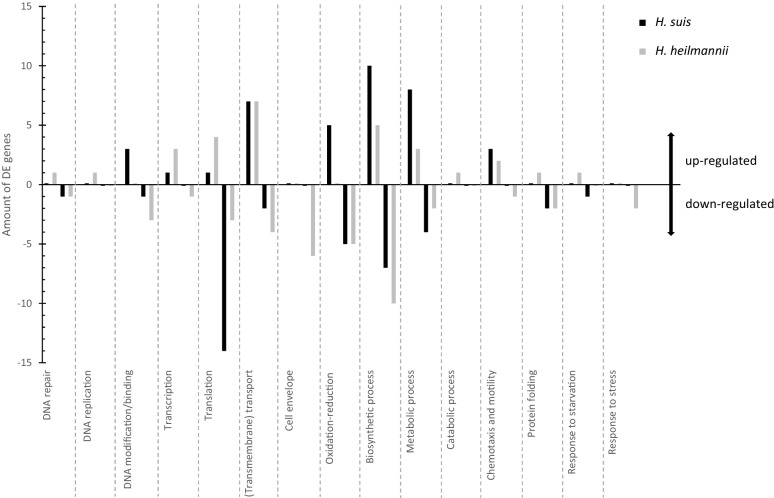
**Classification by function of*H. suis*and*H. heilmannii*differentially expressed (DE) genes.**

The differentially expressed genes in *H. suis* and *H. heilmannii* upon adherence to MKN7 cells compared to unbound bacteria were also classified by function, as shown in Figure 6 and Additional files 11, 12, 13, 14. In general, *H. suis* and *H. heilmannii* had both up- and down-regulated genes within the same functional class (Figure 6). However, the up-regulation of DNA replication related genes and down-regulation of cell envelope related genes and genes involved in response to stress were unique for *H. heilmannii*.

### Distinct gene expression patterns associated with virulence and colonization capacity in *H. suis* and *H. heilmannii* adhering to MKN7 cells

Subsequently, when comparing the up- and down-regulated genes of *H. suis* and *H. heilmannii* upon adhesion to MKN7 cells, and more specifically differentially expressed genes implicated in bacterium–host interactions, several differences between both species were found and described below (Figure 2 and Additional files 1, 2, 3, 4).

#### Ure *genes &* nikB

The urease protein is encoded by two urease subunit genes (i.e. *UreA* and *UreB*) and several urease accessory genes (i.e. *UreE*, *UreF*, *UreG*, and *UreH*) which are necessary for enzyme activity and thus survival of *Helicobacter* in the acidic pH of the stomach [32, 33]. Here, *UreF* was up-regulated in both *H. suis* and *H. heilmannii* (fold change of 2.74 and 2.73, respectively), whereas *UreB* was up-regulated in *H. heilmannii* (fold change of 2.18) but down-regulated (fold change of 2.0) in *H. suis*. Furthermore, *UreH* was uniquely up-regulated (fold change of 2.05) in *H. suis*. In addition, urease requires nickel for enzyme activity [34]. In our study, the nickel transport system permease protein gene (*NikB*) was significantly upregulated in *H. suis* (fold change of 2.03), but not in *H. heilmannii* upon adhesion to MKN7 cells.

#### *Flagella encoding genes*

*Helicobacter suis* and *H. heilmannii* are spiral shaped bacteria containing 4 to 10 bipolar flagella, enabling them to move to and into the mucus layer of the gastric epithelium [8, 35]. *Helicobacter* movement is mainly driven by chemotaxis, whereby pH, CO_2_, arginine, urea/NH_4_, and molecules that lead to bacterial energy generation are detected [36]. Two genes involved in chemotaxis (i.e. the methyl-accepting chemotaxis protein 4 gene and the methyl-accepting chemotaxis protein gene (*PctC*)) and 1 gene encoding a flagellar basal body P-ring biosynthesis protein (*FlgA*) were significantly up-regulated in *H. suis* (fold changes of 3.65, 2.30, and 3.13, respectively) upon binding to MKN7 cells, whereas 2 flagella related genes (flagellar assembly factor gene (*FliW*) and a gene encoding a secreted protein involved in flagellar motility) were significantly up-regulated in *H. heilmannii* (fold changes of 2.20 and 2.86, respectively). Remarkably, an *FliW* homolog gene was significantly down-regulated in *H. heilmannii* (fold change of 3.01) when bound to the human gastric epithelial cells.

#### Tipα

Tumor necrosis factor-α inducing protein (Tipα) is considered to be involved in carcinogenesis as well as in colonization of the gastric mucosa by *Helicobacter* [37–39]. In our study, *Tipα* was significantly up-regulated (fold change of 2.59) in *H. suis*, but not in *H. heilmannii*.

#### Ggt

The gamma-glutamyl transpeptidase (GGT), contributing to gastric colonization, inhibition of T-cell proliferation, and gastric epithelial cell death, was significantly up-regulated in *H. heilmannii* (fold change of 2.17), but not in *H. suis* upon binding to the gastric MKN7 cells [40–42].

#### *OMPs*

*Helicobacter* species contain a large set of outer membrane proteins (OMPs) as an adaptation to the hostile environment of the stomach [43]. Their outer membrane proteome takes an important role in the colonization of the gastric mucosa. Several outer membrane protein (OMP) genes (*omp4*, *omp13*, *omp27*, *omp30*, and 1 putative *omp* gene) were significantly down-regulated (fold changes of 2.46, 2.11, 2.22, 5.26, and 2.58, respectively) in *H. heilmannii*, but not in *H. suis*.

#### Ppi

A gene encoding peptidyl-prolyl *cis,trans*-isomerase (i.e. *ppi*), which might play a role in gastric inflammation and apoptosis of gastric epithelial cells, was significantly down-regulated (fold change of 2.53) in *H. heilmannii* but not in *H. suis* upon binding to MKN7 cells [44].

## Discussion

The human gastric epithelial MKN7 cell line, derived from a well differentiated gastric tubular adenocarcinoma, was used as it resembles a normal, polarized gastric epithelium with mucin production [19, 20]. Nevertheless, keeping in mind the neoplastic background, the effects of receptor phenotypes on *H. suis* and *H. heilmannii* could be different from the effects of either in vivo gastric cells or primary cell lines [13]. Therefore, potential differences in gene regulation of adherent *H. suis* and *H. heilmannii* might appear when comparing our results with a healthy, non-neoplastic gastric mucosa in vivo.

Quantitative fluorescence-based adherence assays demonstrated an equal, albeit low, binding capacity of the *H. suis* and *H. heilmannii* strains to human gastric epithelial cells. In contrast with *H. pylori*, humans are not commonly infected with *H. suis* and *H. heilmannii*, which may have contributed to the low binding capacity observed in this study. For example, it is possible that non-natural host cells lack essential receptors for *H. suis* and *H. heilmannii*. Still, the use of human gastric epithelial cells in our study was justified since we were particularly interested in the pathogenesis of *H. suis* and *H. heilmannii* in human patients, and less in their natural host.

The low binding level of *H. suis* and *H. heilmannii* was translated in a low percentage of identified reads that mapped against the *H. suis* and *H. heilmannii* genome. Despite this low percentage, it was justified to further analyze the differential expression of the identified features as sequencing depth was shown to be good. Saturation curves reached a near horizontal plateau, indicating that the gain of new features was small compared to the number of added reads. Since the case groups in our study had almost two orders of magnitude less reads compared to controls, we used DESeq2 as a statistical model as it is claimed to be somewhat more robust when library sizes are very different [45].

Reciprocal BLASTp analysis showed only 2 commonly up-regulated and 4 commonly down-regulated genes among the *H. suis* and *H. heilmannii* strains tested here. The low correspondence in differentially expressed genes among both *Helicobacter* species indicates a distinct transcriptome of the adherent *H. suis* and *H. heilmannii* strains. Furthermore, 2 down-regulated *H. suis* genes (i.e. *S10* and *UreB*) had an up-regulated *H. heilmannii* homolog. These opposite gene regulations in the *H. suis* and *H. heilmannii* strains demonstrated in vitro might indicate that different virulence-associated factors are involved in the pathogenesis of both *Helicobacter* species upon adherence to the human gastric epithelium in vivo.

When classifying the differentially expressed *H. suis* and *H. heilmannii* genes according to their function, it is clear that genes of many different functional classes are expressed upon contact of the bacteria with the human gastric epithelium. The involvement of diverse functional classes upon adhesion was also described for *H. pylori* and indicates that adhesion to the human gastric epithelium initiates pleiotropic adaptive responses in the bacterium [13]. Discrepancies in functional classes, but also at individual gene expression level between the *H. suis* and *H. heilmannii* strains in vitro further underline the probability of distinct pathogenic pathways between both species upon adherence to the human gastric mucosa in vivo.

In general, prediction of the function of *H. suis* and *H. heilmannii* genes is based on extrapolation of what is known for *H. pylori* [24, 46]. It would have been interesting to have included a *H. pylori* strain in this study, as it would allow comparison of gene regulation of *H. suis* and *H. heilmannii* with *H. pylori*. Still, several genes associated with gastric colonization and virulence of *H. pylori* were differentially expressed in *H. suis* and *H. heilmannii* upon binding to MKN7 cells, indicating that the pathogenesis of animal-associated *Helicobacter* spp. infections clearly differs from *H. pylori*.

Gastric *Helicobacter* species produce urease for their survival and colonization in the acidic environment of the stomach. Urease is a multi-subunit complex which hydrolyzes host-derived urea to ammonia and carbonic acid. The released ammonia neutralizes stomach HCl, creating a neutral microenvironment around the bacterium [32, 47]. The metallocenter of urease consists of two Ni^2+^ ions, which is important for urease enzyme activity [34]. In our study, *UreB* and *UreF* were differentially expressed in adherent *H. suis* and *H. heilmannii*, but expression of *UreH* and *NikB* was only altered in adherent *H. suis*. In *H. pylori*, the function of *UreF* is still unknown, whereas *UreH* probably facilitates proper assembly of the urease metallocenter [48]. Interestingly, changes in expression of urease gene subunits have not been described for *H. pylori* upon attachment to human gastric adenocarcinoma cell lines [13]. These in vitro findings may indicate that urease activity is differently regulated among different *Helicobacter* species when adhering to human gastric epithelium in vivo.

Apart from urease, motility and chemotaxis are essential for gastric *Helicobacter* colonization and persistence [49, 50]. Upon adhesion to human gastric epithelial cells, *FlgA*, *PctC*, and a methyl-accepting chemotaxis protein 4 gene of *H. suis* and *FliW* and a gene encoding a secreted protein involved in flagellar motility of *H. heilmannii* were significantly up-regulated. Additionally, a *FliW* homolog was down-regulated in adherent *H. heilmannii*. In *H. pylori*, however, a different set of genes encoding flagellar motility have been shown to be up-regulated (i.e. *flgB*) or down-regulated (i.e. *flgM*, *flgG*, and *flaA*) upon adherence to epithelial cells [13]. These results reinforce previous observations that *Helicobacter* species present different mechanisms of chemotaxis and motility regulation [51].

Another virulence factor is the tumor necrosis factor-α inducing protein (Tipα). This protein binds to cell-surface nucleolin, which transports Tipα into the cytosol and nuclei of gastric cells, thereby activating NFκB and inducing the expression of TNF-α, IL-1β, IL-8, the up-regulation of *Bcl*-*2* and the down-regulation of *p53* [38, 39]. Besides its carcinogenic role, Tipα is also considered to be involved in the stimulation of macrophages and colonization of the gastric mucosa by *Helicobacter* spp. [37]. Based on our results, the *H. suis* and *H. heilmannii* strains used here are able to colonize the human gastric epithelium in similar levels (Figure 1). However, *Tipα* was only significantly up-regulated in the *H. suis* strain, indicating that gastric colonization of the *H. heilmannii* strain is probably regulated by other genes.

Gamma-glutamyl transpeptidase (GGT) has been described to promote gastric inflammation, to contribute to peptic ulcer development, to play a role in the colonization of the gastric mucosa and to modulate the host immune response [42, 52]. Since *ggt* was only up-regulated in the *H. heilmannii* strain upon adherence, this enzyme could represent an essential factor for human gastric colonization by *H. heilmannii*. Previously, it was demonstrated that *H. suis ggt* mutant strains were capable of colonizing the stomach at levels comparable to *H. suis* wild type strains [53], confirming that GGT is not essential for *H. suis* colonization. The role of GGT in *H*. *pylori* colonization remains inconclusive as no differences in *ggt* gene expression were shown upon binding to epithelial cells [13], although an impaired colonization capacity of a *H. pylori ggt* mutant strain was demonstrated in mice [53–55].

Bacterial OMPs are directly involved in the interactions of pathogenic bacteria with their environment. However, the role of the different *H. suis* and *H. heilmannii* OMPs in gastric colonization and pathogenesis remains largely unknown [43]. While *H. suis* and *H. heilmannii* lack all known *H. pylori* adhesins described so far [24, 46, 56], *H. heilmannii* HofE and HofF OMPs have been shown to be important for adhesion to the mouse gastric mucosa, with a higher affinity for gastric epithelial cells than for mucins [57]. Furthermore, two OMP families (i.e. the eight-stranded β-barrel outer membrane protein W (ompW) family and the *Borrelia burgdorferi* outer membrane spanning (BP-oms28) porin family) seem to be uniquely present in *H. suis* [43]. In our study, no significant changes in expression could be demonstrated neither for the *H. heilmannii HofE* and *HofF* genes, nor for members of the ompW and BP-oms28 family of *H. suis*. However, several other OMP genes were significantly down-regulated upon MKN7 cell adhesion in the *H. heilmannii* strain, but not in the *H. suis* strain. Such down-regulation of OMP genes has also been demonstrated in *H. pylori* [13] and might be explained by bacterial cell wall modification as a result of proximity and binding of the bacteria to the host cells. For the *H. suis* strain, no OMP genes were differentially expressed upon epithelial cell adhesion, which suggests a difference in OMP regulation between *H. suis* and *H. heilmannii* upon human gastric colonization.

A final virulence factor which was differentially expressed in the *H. heilmannii* strain upon adherence to human gastric epithelial cells was the secreted peptidyl-prolyl *cis,trans*-isomerase (PPI). In *H. pylori*, this protein is known to induce IL-6 release from macrophages and to induce apoptosis of gastric epithelial cells by a cascade of mechanisms initiated through its interaction with toll-like receptor 4 [44]. Indeed, mutant *H. pylori* strains with inactivated PPI have a very low apoptosis-inducing capacity [44]. The down-regulation of the PPI-encoding gene in the *H. heilmannii* strain, but not in *H. suis* strain, might indicate that *H. heilmannii* is less likely to induce apoptosis of gastric epithelial cells compared to *H. suis*. However, the role of PPI in *H. suis* and *H. heilmannii* needs further elucidation.

Taken together, a large set of *H. suis* and *H. heilmannii* genes were significantly up-regulated or down-regulated upon adhesion to human gastric epithelial MKN7 cells. Genes with altered expression profiles belonged to many different functional classes and only few genes were commonly up-regulated or down-regulated in both *H. suis* and *H. heilmannii*, indicating that these pathogens follow distinct pathways upon adherence to the human gastric epithelium in vivo.

To further analyze the role of these differentially expressed genes in human gastric colonization and virulence, *H. suis* and *H. heilmannii* mutants lacking these genes should be created and tested using in vitro and in vivo models.

## Supplementary information

**Additional file 1. List of 83 significantly up-regulated** ***H. suis*** **genes in cases compared to controls (with p**_**adj**_ **≤ 0.01; fold change ≥ 2).**

**Additional file 2. List of 51 significantly down-regulated** ***H. suis*** **genes in cases compared to controls (with p**_**adj**_ **≤ 0.01; fold change ≤ -2).**

**Additional file 3. List of 60 significantly up-regulated** ***H. heilmannii*** **genes in cases compared to controls (with p**_**adj**_ **≤ 0.01; fold change ≥ 2).**

**Additional file 4. List of 83 significantly down-regulated** ***H. heilmannii*** **genes in cases compared to controls (with p**_**adj**_ **≤ 0.01; fold change ≤ -2).**

**Additional file 5. Volcano plots of the comparisons (case versus control).** Red dots represent significantly differentially expressed genes (p_adj_ ≤ 0.01, fold change ≥ 2) in MKN7-bound helicobacters (i.e. cases) compared to unbound helicobacters (i.e. controls). (A) Volcano plot of the comparison (case versus control) for *H. suis*. (B) Volcano plot of the comparison (case versus control) for *H. heilmannii.*

**Additional file 6. Plots of the principal component analysis (PCA) of the restricted set of differentially expressed features.** First 2 components (PC1 and PC2) of a PCA, with percentages of variance associated with each axis. These plots show the separation between samples based on the main sources of variation found in the rlog-transformed data sets (p_adj_ ≤ 0.01, fold change ≥ 2). (A) PCA-plot for *H. suis* (134 differentially expressed features) (B) PCA-plot for *H. heilmannii* (143 differentially expressed features). Blue dots indicate control samples (i.e. unbound *Helicobacter*); red dots indicate cases (i.e. *Helicobacter* bound to MKN7 cells). In both PCA plots, the sample groups (i.e. case and control) are well separated, indicating that the main variability within each experiment came from biological differences between cases and controls.

**Additional file 7.** ***H. suis*** **up-regulated genes with (72) and without (11)** ***H. heilmannii*** **homologs according to BLASTp.**

**Additional file 8.** ***H. suis*** **down-regulated genes with (45) and without (6)** ***H. heilmannii*** **homologs according to BLASTp.**

**Additional file 9.** ***H. heilmannii*** **up-regulated genes with (47) and without (13)** ***H. suis*** **homologs according to BLASTp.**

**Additional file 10.** ***H. heilmannii*** **down-regulated genes with (74) and without (9)** ***H. suis*** **homologs according to BLASTp.**

**Additional file 11. Classification of up-regulated** ***H. suis*** **genes in cases compared to controls according to their function.**

**Additional file 12. Classification of up-regulated** ***H. heilmannii*** **genes in cases compared to controls according to their function.**

**Additional file 13. Classification of down-regulated** ***H. suis*** **genes in cases compared to controls according to their function.**

**Additional file 14. Classification of down-regulated** ***H. heilmannii*** **genes in cases compared to controls according to their function.**
