# Supplementary material for: Distinct transcriptome signatures of Helicobacter suis and Helicobacter heilmannii strains upon adherence to human gastric epithelial cells
Source: Vet Res. 2020 May 7;51:62. doi: 10.1186/s13567-020-00786-w (PMC7206758; doi:10.1186/s13567-020-00786-w)
Supplement: Supplementary file 1 — Additional file 1. List of 83 significantly up-regulated H. suis genes in cases compared to controls (with p adj ≤ 0.01; fold change ≥ 2). [file 13567_2020_786_MOESM1_ESM.docx]

| **Id** | **Description** | **Biological process** | **Molecular function** | **Fold change** | ***p*-value** | **p_adj_** |
| --- | --- | --- | --- | --- | --- | --- |
| 104628.16_00453 | hypothetical protein |  |  | 5.563 | 4.02E-20 | 2.89E-18 |
| 104628.16_01470 | hypothetical protein |  |  | 4.675 | 1.69E-18 | 8.93E-17 |
| 104628.16_01297 | Phosphorylase superfamily protein | nucleoside metabolic process | catalytic activity | 3.893 | 1.63E-12 | 5.04E-11 |
| 104628.16_00432 | hypothetical protein |  |  | 3.783 | 2.79E-07 | 3.12E-06 |
| 104628.16_01371 | Methyl-accepting chemotaxis protein 4 | chemotaxis, | transmembrane signaling receptor activity | 3.645 | 1.60E-20 | 1.33E-18 |
|  |  | signal transduction |  |  |  |  |
| 104628.16_00341 | hypothetical protein |  |  | 3.632 | 1.09E-19 | 6.91E-18 |
| 104628.16_01410 | DNA methylase | DNA methylation, | DNA binding, N-methyltransferase activity, | 3.617 | 2.04E-05 | 1.52E-04 |
|  |  | N-4 methylation of cytosine | site-specific DNA-methyltransferase (cytosine-N4-specific) activity |  |  |  |
| 104628.16_00573 | hypothetical protein |  |  | 3.566 | 2.24E-35 | 1.18E-32 |
| 104628.16_01441 | Helix-turn-helix domain protein |  |  | 3.336 | 1.28E-07 | 1.59E-06 |
| 104628.16_01509 | hypothetical protein |  |  | 3.309 | 1.78E-04 | 1.00E-03 |
| 104628.16_01010 | Acyl-CoA thioester hydrolase YbgC |  | hydrolase activity | 3.304 | 2.13E-07 | 2.51E-06 |
| 104628.16_01096 | flagellar basal body P-ring biosynthesis protein FlgA |  |  | 3.133 | 2.05E-05 | 1.52E-04 |
| 104628.16_01539 | CDP-diacylglycerol--glycerol-3-phosphate 3-phosphatidyltransferase | phospholipid biosynthetic process | CDP-diacylglycerol-glycerol-3-phosphate 3-phosphatidyltransferase activity, phosphotransferase activity for other substituted phosphate groups | 3.127 | 2.73E-04 | 1.45E-03 |
| 104628.16_00173 | Mechanosensitive ion channel | transmembrane transport |  | 3.096 | 7.87E-26 | 1.13E-23 |
| 104628.16_01099 | Thymidylate kinase | dTDP biosynthetic process | thymidylate kinase activity, | 3.019 | 2.59E-04 | 1.39E-03 |
|  |  |  | ATP binding |  |  |  |
| 104628.16_00123 | hypothetical protein |  |  | 2.947 | 4.70E-04 | 2.31E-03 |
| 104628.16_00037 | hypothetical protein |  |  | 2.873 | 3.89E-07 | 4.14E-06 |
| **Id** | **Description** | **Biological process** | **Molecular function** | **Fold change** | ***p*-value** | **p_adj_** |
| 104628.16_00761 | Ribosomal large subunit pseudouridine synthase D | pseudouridine synthesis,  RNA modification | RNA binding,  pseudouridine synthase activity | 2.871 | 5.64E-04 | 2.70E-03 |
| 104628.16_01608 | hypothetical protein |  |  | 2.871 | 1.37E-06 | 1.31E-05 |
| 104628.16_00558 | hypothetical protein |  |  | 2.839 | 1.63E-06 | 1.51E-05 |
| 104628.16_01370 | CobQ/CobB/MinD/ParA nucleotide binding domain protein |  |  | 2.831 | 1.73E-06 | 1.58E-05 |
| 104628.16_00561 | hypothetical protein |  |  | 2.824 | 1.25E-09 | 2.42E-08 |
| 104628.16_00837 | hypothetical protein |  |  | 2.797 | 1.11E-28 | 2.20E-26 |
| 104628.16_00395 | Urease accessory protein UreF | nitrogen compound metabolic process | nickel cation binding | 2.737 | 8.56E-13 | 2.73E-11 |
| 104628.16_01497 | NAD(P)H-quinone oxidoreductase subunit 3 | oxidation-reduction process | NADH dehydrogenase (ubiquinone) activity | 2.707 | 7.64E-08 | 1.07E-06 |
| 104628.16_01165 | putative copper-transporting ATPase PacS | cation transport, metal ion transport | nucleotide binding,  cation-transporting ATPase activity,  metal ion binding | 2.701 | 1.25E-14 | 4.83E-13 |
| 104628.16_01201 | Putative phosphoribosyl transferase/MT0597 | nucleoside metabolic process |  | 2.681 | 6.37E-07 | 6.56E-06 |
| 104628.16_01171 | hypothetical protein |  |  | 2.618 | 4.08E-04 | 2.02E-03 |
| 104628.16_00302 | Oligopeptide-binding protein AppA precursor | transmembrane transport |  | 2.600 | 7.50E-22 | 7.42E-20 |
| 104628.16_00174 | tumor necrosis factor alpha-inducing protein |  |  | 2.590 | 1.48E-100 | 2.35E-97 |
| 104628.16_01508 | hypothetical protein |  |  | 2.574 | 1.04E-03 | 4.65E-03 |
| 104628.16_01489 | NADH-quinone oxidoreductase  subunit J | oxidation-reduction process | NADH dehydrogenase (ubiquinone) activity | 2.568 | 6.19E-09 | 1.07E-07 |
| 104628.16_00443 | hypothetical protein |  |  | 2.520 | 1.18E-06 | 1.15E-05 |
| 104628.16_01569 | paraquat-inducible protein B |  |  | 2.513 | 4.62E-15 | 1.93E-13 |
| **Id** | **Description** | **Biological process** | **Molecular function** | **Fold change** | ***p*-value** | **p_adj_** |
| 104628.16_01030 | Transaldolase | pentose-phosphate shunt carbohydrate metabolic process | catalytic activity,  sedoheptulose-7-phosphate:D-glyceraldehyde-3-phosphate glyceronetransferase activity | 2.495 | 6.40E-20 | 4.41E-18 |
| 104628.16_01363 | Glycosyltransferase family 9 (heptosyltransferase) | metabolic process | transferase activity,  transferring glycosyl groups | 2.473 | 3.65E-04 | 1.85E-03 |
| 104628.16_01614 | hypothetical protein |  |  | 2.465 | 1.62E-03 | 6.76E-03 |
| 104628.16_01437 | hypothetical protein |  |  | 2.432 | 1.96E-03 | 8.04E-03 |
| 104628.16_00791 | hypothetical protein |  |  | 2.423 | 9.86E-11 | 2.44E-09 |
| 104628.16_00868 | preprotein translocase subunit SecF | intracellular protein transport | P-P-bond-hydrolysis-driven protein transmembrane transporter activity | 2.422 | 1.50E-05 | 1.14E-04 |
| 104628.16_00843 | Amidophosphoribosyltransferase precursor | nucleoside metabolic process |  | 2.385 | 1.06E-05 | 8.31E-05 |
| 104628.16_01404 | putative inorganic polyphosphate/ATP-NAD kinase | NADP biosynthetic process, metabolic process, NAD metabolic process | NAD+ kinase activity | 2.382 | 1.72E-04 | 9.79E-04 |
| 104628.16_01324 | D-glycero-alpha-D-manno-heptose-1,7-bisphosphate 7-phosphatase | carbohydrate metabolic process | phosphatase activity | 2.363 | 5.85E-08 | 8.34E-07 |
| 104628.16_01557 | L-seryl-tRNA(Sec) selenium transferase | selenocysteine incorporation | catalytic activity,  L-seryl-tRNASec selenium transferase activity | 2.363 | 2.42E-09 | 4.41E-08 |
| 104628.16_00465 | hypothetical protein |  |  | 2.362 | 7.95E-05 | 4.96E-04 |
| 104628.16_01335 | hypothetical protein |  |  | 2.344 | 1.86E-14 | 6.84E-13 |
| 104628.16_01546 | Fic/DOC family protein |  |  | 2.341 | 4.17E-04 | 2.06E-03 |
| 104628.16_01347 | hypothetical protein |  |  | 2.322 | 3.55E-12 | 1.00E-10 |
| 104628.16_01189 | Octaprenyl-diphosphate synthase | isoprenoid biosynthetic process |  | 2.313 | 2.23E-11 | 5.90E-10 |
| 104628.16_01061 | Methyl-accepting chemotaxis protein PctC |  |  | 2.302 | 6.56E-05 | 4.17E-04 |
| 104628.16_01560 | putative undecaprenyl-phosphate N-acetylglucosaminyl 1-phosphate transferase |  | phospho-N-acetylmuramoyl-pentapeptide-transferase activity | 2.300 | 1.29E-04 | 7.56E-04 |
| 104628.16_00004 | Ferrochelatase | heme biosynthetic process | ferrochelatase activity | 2.293 | 3.75E-04 | 1.89E-03 |
| 104628.16_00165 | hypothetical protein |  |  | 2.284 | 1.46E-06 | 1.38E-05 |
| 104628.16_01022 | ABC-2 family transporter protein |  |  | 2.283 | 6.23E-15 | 2.53E-13 |
| **Id** | **Description** | **Biological process** | **Molecular function** | **Fold change** | ***p*-value** | **p_adj_** |
| 104628.16_00759 | Putative nickel-responsive regulator | regulation of transcription,  DNA-templated,  response to nickel cation | DNA binding,  nickel cation binding | 2.278 | 5.62E-05 | 3.66E-04 |
| 104628.16_00811 | hypothetical protein |  |  | 2.273 | 6.22E-06 | 5.24E-05 |
| 104628.16_01166 | tRNA1(Val) (adenine(37)-N6)-methyltransferase | methylation | nucleic acid binding, methyltransferase activity | 2.270 | 2.28E-03 | 9.13E-03 |
| 104628.16_00145 | 3-phosphoshikimate 1-carboxyvinyltransferase | aromatic amino acid family biosynthetic process | catalytic activity,  3-phosphoshikimate 1-carboxyvinyltransferase activity,  transferase activity,  transferring alkyl or aryl (other than methyl) groups | 2.269 | 4.33E-09 | 7.62E-08 |
| 104628.16_00999 | hypothetical protein |  |  | 2.260 | 1.20E-03 | 5.26E-03 |
| 104628.16_01571 | putative phospholipid ABC transporter permease protein MlaE | transport |  | 2.247 | 3.15E-04 | 1.64E-03 |
| 104628.16_01211 | hypothetical protein |  |  | 2.242 | 2.47E-06 | 2.18E-05 |
| 104628.16_00480 | hypothetical protein |  |  | 2.239 | 1.66E-05 | 1.25E-04 |
| 104628.16_01253 | 3-deoxy-manno-octulosonate cytidylyltransferase |  | 3-deoxy-manno-octulosonate cytidylyltransferase activity | 2.228 | 2.32E-09 | 4.28E-08 |
| 104628.16_01504 | hypothetical protein |  |  | 2.226 | 1.30E-03 | 5.58E-03 |
| 104628.16_01377 | 30S ribosomal protein S20 | translation | RNA binding,  structural constituent of ribosome | 2.216 | 2.96E-08 | 4.47E-07 |
| 104628.16_01310 | Soluble lytic murein transglycosylase precursor |  |  | 2.208 | 2.94E-07 | 3.23E-06 |
| 104628.16_00789 | Farnesyl diphosphate synthase | isoprenoid biosynthetic process |  | 2.203 | 2.96E-11 | 7.69E-10 |
| 104628.16_00764 | GTP cyclohydrolase 1 type 2 |  |  | 2.194 | 1.71E-06 | 1.58E-05 |
| 104628.16_00906 | hypothetical protein |  |  | 2.190 | 1.55E-03 | 6.53E-03 |
| 104628.16_01120 | PD-(D/E)XK nuclease superfamily protein |  |  | 2.175 | 4.41E-33 | 1.40E-30 |
| 104628.16_00201 | hypothetical protein |  |  | 2.171 | 3.81E-04 | 1.90E-03 |
| 104628.16_00348 | ribonuclease BN/unknown domain fusion protein |  |  | 2.169 | 4.21E-08 | 6.23E-07 |
| **Id** | **Description** | **Biological process** | **Molecular function** | **Fold change** | ***p*-value** | **p_adj_** |
| 104628.16_01380 | hypothetical protein |  |  | 2.107 | 6.57E-09 | 1.12E-07 |
| 104628.16_00648 | hypothetical protein |  |  | 2.090 | 3.68E-04 | 1.86E-03 |
| 104628.16_00482 | putative oxidoreductase YdgJ |  | oxidoreductase activity | 2.073 | 2.34E-03 | 9.28E-03 |
| 104628.16_00438 | DNA primase |  | DNA replication,  synthesis of RNA primer | 2.059 | 5.63E-06 | 4.77E-05 |
| 104628.16_00393 | Urease accessory protein UreH | nitrogen compound metabolic process | nickel cation binding | 2.051 | 1.07E-10 | 2.60E-09 |
| 104628.16_00450 | Ribosomal protein S12 methylthiotransferase RimO | tRNA modification,  peptidyl-L-beta-methylthioaspartic acid biosynthetic process from peptidyl-aspartic acid | catalytic activity,  transferase activity,  iron-sulfur cluster binding,  4 iron, 4 sulfur cluster binding | 2.045 | 3.43E-23 | 3.88E-21 |
| 104628.16_01486 | NAD(P)H-quinone oxidoreductase chain 4 1 | ATP synthesis coupled electron transport, oxidation-reduction process | NADH dehydrogenase (ubiquinone) activity | 2.035 | 5.77E-08 | 8.30E-07 |
| 104628.16_00362 | hypothetical protein |  |  | 2.029 | 1.07E-06 | 1.06E-05 |
| 104628.16_00303 | Nickel transport system permease protein NikB | transmembrane transport |  | 2.028 | 8.87E-04 | 4.05E-03 |
| 104628.16_00155 | Fumarate reductase cytochrome b subunit | tricarboxylic acid cycle | oxidoreductase activity, acting on the CH-CH group of donors | 2.013 | 7.22E-10 | 1.43E-08 |
| 104628.16_00717 | hypothetical protein |  |  | 2.001 | 1.31E-03 | 5.63E-03 |
