# Supplementary material for: Distinct transcriptome signatures of Helicobacter suis and Helicobacter heilmannii strains upon adherence to human gastric epithelial cells
Source: Vet Res. 2020 May 7;51:62. doi: 10.1186/s13567-020-00786-w (PMC7206758; doi:10.1186/s13567-020-00786-w)
Supplement: Supplementary file 2 — Additional file 2. List of 51 significantly down-regulated H. suis genes in cases compared to controls (with p adj ≤ 0.01; fold change ≤ -2). [file 13567_2020_786_MOESM2_ESM.docx]

| **Id** | **Description** | **Biological process** | **Molecular function** | **Fold change** | ***p*-value** | **p_adj_** |
| --- | --- | --- | --- | --- | --- | --- |
| 104628.16_00398 | Urease subunit beta | nitrogen compound metabolic process | urease activity,  nickel cation binding,  hydrolase activity,  acting on carbon-nitrogen (but not peptide) bonds | -2.004 | 1.28E-25 | 1.69E-23 |
| 104628.16_01444 | hypothetical protein |  |  | -2.004 | 1.56E-06 | 1.46E-05 |
| 104628.16_01111 | General stress protein 16U | response to stress |  | -2.053 | 9.18E-09 | 1.50E-07 |
| 104628.16_00154 | Fumarate reductase flavoprotein subunit | electron transport chain,  oxidation-reduction process | oxidoreductase activity,  acting on the CH-CH group of donors,  flavin adenine dinucleotide binding | -2.066 | 1.03E-14 | 4.08E-13 |
| 104628.16_00033 | hypothetical protein |  |  | -2.079 | 1.69E-10 | 4.00E-09 |
| 104628.16_00458 | hypothetical protein |  |  | -2.079 | 2.24E-03 | 9.02E-03 |
| 104628.16_00356 | recombinase A | DNA metabolic process,  DNA repair | DNA binding,  single-stranded DNA binding,  ATP binding,  DNA-dependent ATPase activity | -2.092 | 1.19E-07 | 1.52E-06 |
| 104628.16_01554 | N-carbamoyl-D-amino acid hydrolase | nitrogen compound metabolic process |  | -2.105 | 1.73E-04 | 9.79E-04 |
| 104628.16_00747 | Elongation factor G | translational elongation | translation elongation factor activity,  GTPase activity,  GTP binding | -2.110 | 2.18E-30 | 5.75E-28 |
| 104628.16_00529 | putative FAD-linked oxidoreductase | oxidation-reduction process | catalytic activity,  glycolate oxidase activity,  oxidoreductase activity,  acting on CH-OH group of donors,  flavin adenine dinucleotide binding | -2.128 | 8.16E-06 | 6.59E-05 |
| **Id** | **Description** | **Biological process** | **Molecular function** | **Fold change** | ***p*-value** | **p_adj_** |
| 104628.16_00651 | heat shock protein GrpE | protein folding | adenyl-nucleotide exchange factor activity,  protein homodimerization activity, chaperone binding | -2.128 | 2.98E-05 | 2.13E-04 |
| 104628.16_00282 | Acyl carrier protein | fatty acid biosynthetic process |  | -2.146 | 9.32E-04 | 4.20E-03 |
| 104628.16_01600 | Phospho-2-dehydro-3-deoxyheptonate aldolase | aromatic amino acid family biosynthetic process | catalytic activity,  3-deoxy-7-phosphoheptulonate synthase activity | -2.174 | 1.04E-05 | 8.22E-05 |
| 104628.16_00269 | UDP-2-acetamido-3-amino-2,3-dideoxy-D-glucuronate N-acetyltransferase | / | transferase activity | -2.193 | 4.33E-05 | 2.94E-04 |
| 104628.16_00222 | 50S ribosomal protein L1 | translation | RNA binding,  structural constituent of ribosome | -2.237 | 1.57E-08 | 2.49E-07 |
| 104628.16_00967 | Ubiquinol-cytochrome c reductase iron-sulfur subunit | oxidation-reduction process | oxidoreductase activity,  acting on diphenols and related substances as donors,  2 iron, 2 sulfur cluster binding | -2.247 | 1.17E-04 | 7.02E-04 |
| 104628.16_00440 | 60 kDa chaperonin 1 | protein folding, protein refolding | ATP binding | -2.283 | 1.72E-20 | 1.36E-18 |
| 104628.16_00313 | Dipeptide transport system permease protein DppC | transmembrane transport |  | -2.288 | 1.55E-06 | 1.46E-05 |
| 104628.16_00429 | Acetophenone carboxylase delta subunit |  | catalytic activity | -2.288 | 1.12E-03 | 4.92E-03 |
| 104628.16_00081 | Modification methylase DpnIIB | DNA methylation, methylation | nucleic acid binding,  DNA binding,  methyltransferase activity,  N-methyltransferase activity | -2.304 | 6.64E-06 | 5.54E-05 |
| 104628.16_00896 | ATP synthase gamma chain | ATP synthesis coupled proton transport | proton-transporting ATP synthase activity,  rotational mechanism | -2.304 | 2.73E-14 | 9.83E-13 |
| 104628.16_00032 | hypothetical protein |  |  | -2.353 | 1.40E-34 | 5.53E-32 |
| 104628.16_00500 | 50S ribosomal protein L4 | translation | structural constituent of ribosome | -2.364 | 1.94E-06 | 1.76E-05 |
| 104628.16_00505 | 30S ribosomal protein S3 | translation | nucleic acid binding, RNA binding, structural constituent of ribosome | -2.364 | 2.85E-10 | 6.19E-09 |
| 104628.16_00515 | 50S ribosomal protein L18 | translation | structural constituent of ribosome | -2.364 | 6.81E-09 | 1.14E-07 |
| 104628.16_00726 | hypothetical protein |  |  | -2.392 | 5.75E-04 | 2.73E-03 |
| 104628.16_00510 | 50S ribosomal protein L24 | translation | structural constituent of ribosome | -2.400 | 1.47E-04 | 8.41E-04 |
| **Id** | **Description** | **Biological process** | **Molecular function** | **Fold change** | ***p*-value** | **p_adj_** |
| 104628.16_00283 | 3-oxoacyl-[acyl-carrier-protein] synthase 2 | fatty acid biosynthetic process, metabolic process | catalytic activity,  transferase activity,  transferring acyl groups other than amino-acyl groups | -2.410 | 1.65E-12 | 5.04E-11 |
| 104628.16_01123 | Thioredoxin reductase | removal of superoxide radicals, oxidation-reduction process | thioredoxin-disulfide reductase activity,  oxidoreductase activity | -2.427 | 9.16E-14 | 3.09E-12 |
| 104628.16_00008 | Glutamate racemase | nitrogen compound metabolic process, metabolic process, peptidoglycan biosynthetic process | glutamate racemase activity,  racemase and epimerase activity,  acting on amino acids and derivatives | -2.445 | 8.01E-04 | 3.69E-03 |
| 104628.16_01385 | hypothetical protein |  |  | -2.463 | 1.24E-03 | 5.40E-03 |
| 104628.16_01071 | NifU-like protein | iron-sulfur cluster assembly | iron ion binding,  iron-sulfur cluster binding | -2.500 | 1.56E-19 | 9.18E-18 |
| 104628.16_00499 | 50S ribosomal protein L3 | translation | structural constituent of ribosome | -2.525 | 1.97E-27 | 3.46E-25 |
| 104628.16_00204 | Rod shape-determining protein MreB | cell morphogenesis |  | -2.577 | 8.14E-11 | 2.08E-09 |
| 104628.16_00514 | 50S ribosomal protein L6 | translation | structural constituent of ribosome,  rRNA binding | -2.611 | 2.04E-13 | 6.73E-12 |
| 104628.16_00506 | 50S ribosomal protein L16 | translation | structural constituent of ribosome,  rRNA binding | -2.695 | 8.07E-06 | 6.56E-05 |
| 104628.16_00498 | 30S ribosomal protein S10 | translation | RNA binding,  structural constituent of ribosome | -2.747 | 4.03E-04 | 2.00E-03 |
| 104628.16_00513 | 30S ribosomal protein S8 | translation | structural constituent of ribosome | -2.825 | 2.48E-07 | 2.87E-06 |
| 104628.16_01454 | Chaperone protein ClpB | protein metabolic process | ATP binding | -2.849 | 3.09E-18 | 1.58E-16 |
| 104628.16_00223 | 50S ribosomal protein L10 | ribosome biogenesis |  | -2.890 | 4.60E-21 | 4.05E-19 |
| 104628.16_00509 | 50S ribosomal protein L14 | translation | structural constituent of ribosome | -2.899 | 3.27E-04 | 1.68E-03 |
| 104628.16_00911 | hypothetical protein |  |  | -3.040 | 8.79E-08 | 1.21E-06 |
| 104628.16_00749 | Alcohol dehydrogenase | oxidation-reduction process | zinc ion binding,  oxidoreductase activity | -3.067 | 1.70E-19 | 9.63E-18 |
| **Id** | **Description** | **Biological process** | **Molecular function** | **Fold change** | ***p*-value** | **p_adj_** |
| 104628.16_00961 | Phosphomethylpyrimidine synthase | thiamine biosynthetic process | carbon-carbon lyase activity,  iron-sulfur cluster binding | -3.067 | 1.56E-19 | 9.18E-18 |
| 104628.16_00424 | hypothetical protein |  |  | -3.145 | 3.13E-04 | 1.63E-03 |
| 104628.16_00960 | hypothetical protein |  |  | -3.165 | 7.75E-08 | 1.08E-06 |
| 104628.16_00290 | Shikimate kinase |  |  | -3.195 | 4.78E-06 | 4.14E-05 |
| 104628.16_00125 | hypothetical protein |  |  | -3.448 | 5.77E-09 | 1.00E-07 |
| 104628.16_01016 | Cell wall-associated hydrolase |  |  | -3.676 | 2.41E-08 | 3.69E-07 |
| 104628.16_00507 | 50S ribosomal protein L29 | translation | structural constituent of ribosome | -3.759 | 1.10E-07 | 1.43E-06 |
| 104628.16_00224 | 50S ribosomal protein L7/L12 | translation | structural constituent of ribosome | -3.922 | 1.09E-08 | 1.76E-07 |
