# Supplementary material for: Distinct transcriptome signatures of Helicobacter suis and Helicobacter heilmannii strains upon adherence to human gastric epithelial cells
Source: Vet Res. 2020 May 7;51:62. doi: 10.1186/s13567-020-00786-w (PMC7206758; doi:10.1186/s13567-020-00786-w)
Supplement: Supplementary file 3 — Additional file 3. List of 60 significantly up-regulated H. heilmannii genes in cases compared to controls (with p adj ≤ 0.01; fold change ≥ 2). [file 13567_2020_786_MOESM3_ESM.docx]

| **Id** | **Description** | **Biological process** | **Molecular function** | **Fold change** | ***p*-value** | **p_adj_** |
| --- | --- | --- | --- | --- | --- | --- |
| BN341_5230 | Beta-1,4-galactosyltransferase |  |  | 4.639 | 7.50E-19 | 2.60E-17 |
| BN341_5220 | Beta-1,4-galactosyltransferase |  |  | 3.945 | 3.50E-17 | 9.10E-16 |
| BN341_18980 | hypothetical protein |  |  | 3.889 | 4.00E-06 | 2.30E-05 |
| BN341_6170 | Iron(III) dicitrate transport protein FecA |  |  | 3.701 | 6.70E-26 | 5.20E-24 |
| BN341_9640 | Foldase protein PrsA precursor |  | peptidyl-prolyl *cis,trans*-isomerase activity | 3.624 | 9.40E-86 | 8.50E-83 |
| BN341_8270 | hypothetical protein |  |  | 3.168 | 1.40E-04 | 5.80E-04 |
| BN341_6880 | A/G-specific adenine glycosylase | DNA repair,  base-excision repair | DNA binding,  catalytic activity | 3.036 | 1.40E-05 | 7.20E-05 |
| BN341_16620 | Membrane proteins related to metalloendopeptidases |  |  | 2.921 | 6.20E-21 | 2.70E-19 |
| BN341_5080 | hypothetical protein |  |  | 2.907 | 1.10E-23 | 6.00E-22 |
| BN341_16960 | RNA polymerase sigma factor RpoD | DNA-templated transcription,  initiation,  regulation of transcription,  DNA-templated | DNA binding,  DNA binding transcription factor activity,  bacterial sigma factor activity | 2.897 | 1.00E-59 | 4.70E-57 |
| BN341_11980 | secreted protein involved in flagellar motility |  |  | 2.857 | 3.80E-12 | 5.50E-11 |
| BN341_3880 | hypothetical protein |  |  | 2.792 | 2.40E-04 | 9.70E-04 |
| BN341_10490 | Replicative DNA helicase | DNA replication | DNA binding,  DNA helicase activity,  ATP binding | 2.784 | 1.70E-10 | 1.80E-09 |
| BN341_17100 | Urease accessory protein UreF | nitrogen compound metabolic process | nickel cation binding | 2.732 | 2.10E-11 | 2.70E-10 |
| BN341_4210 | FIG00710144: hypothetical protein |  |  | 2.574 | 4.00E-14 | 6.90E-13 |
| BN341_10170 | conserved hypothetical protein with DUF394 domain |  |  | 2.564 | 1.20E-49 | 4.50E-47 |
| BN341_8650 | hypothetical protein |  |  | 2.540 | 3.00E-12 | 4.50E-11 |
| BN341_130 | Acriflavin resistance protein / Multidrug efflux system CmeDEF | transmembrane transport | transmembrane transporter activity | 2.536 | 2.80E-04 | 1.10E-03 |
| BN341_12170 | DNA-cytosine methyltransferase |  | methyltransferase activity | 2.520 | 1.00E-08 | 8.70E-08 |
| **Id** | **Description** | **Biological process** | **Molecular function** | **Fold change** | ***p*-value** | **p_adj_** |
| BN341_1080 | Ferrous iron transport protein B | ferrous iron transport | GTP binding,  ferrous iron transmembrane transporter activity | 2.495 | 2.90E-11 | 3.50E-10 |
| BN341_11930 | Seryl-tRNA synthetase | tRNA aminoacylation for protein translation,  seryl-tRNA aminoacylation | nucleotide binding,  aminoacyl-tRNA ligase activity,  serine-tRNA ligase activity,  ATP binding | 2.486 | 4.20E-18 | 1.20E-16 |
| BN341_8050 | Carbon starvation protein A | cellular response to starvation |  | 2.429 | 1.40E-09 | 1.30E-08 |
| BN341_19560 | Ferrochelatase, protoheme ferro-lyase | heme biosynthetic process | ferrochelatase activity | 2.410 | 1.70E-09 | 1.60E-08 |
| BN341_19530 | Transcription termination factor Rho | DNA-templated transcription, termination | nucleic acid binding;  RNA binding,  ATP binding,  RNA-dependent ATPase activity | 2.398 | 2.10E-30 | 2.10E-28 |
| BN341_120 | hypothetical protein |  |  | 2.397 | 1.80E-08 | 1.40E-07 |
| BN341_6220 | UDP-N-acetylglucosamine 4,6-dehydratase |  |  | 2.382 | 2.20E-09 | 2.00E-08 |
| BN341_14880 | hypothetical protein |  |  | 2.360 | 7.30E-13 | 1.20E-11 |
| BN341_9900 | hypothetical protein |  |  | 2.329 | 5.00E-06 | 2.80E-05 |
| BN341_8630 | hypothetical protein |  |  | 2.275 | 1.80E-08 | 1.50E-07 |
| BN341_19710 | Methionyl-tRNA formyltransferase | biosynthetic process,  conversion of methionyl-tRNA to N-formyl-methionyl-tRNA | catalytic activity,  methionyl-tRNA formyltransferase activity,  hydroxymethyl-,  formyl- and related transferase activity | 2.272 | 1.20E-09 | 1.20E-08 |
| BN341_14920 | hypothetical protein |  |  | 2.266 | 5.90E-08 | 4.40E-07 |
| BN341_14970 | hypothetical protein |  |  | 2.243 | 4.10E-08 | 3.10E-07 |
| BN341_19730 | ATP synthase B' chain | ATP synthesis coupled proton transport | proton transmembrane transporter activity | 2.236 | 9.80E-10 | 9.50E-09 |
| BN341_16760 | Acetophenone carboxylase subunit Apc3 |  | hydrolase activity | 2.222 | 8.30E-33 | 9.40E-31 |
| BN341_16830 | Flagellar assembly factor FliW | bacterial-type flagellum assembly |  | 2.202 | 9.20E-08 | 6.60E-07 |
| **Id** | **Description** | **Biological process** | **Molecular function** | **Fold change** | ***p*-value** | **p_adj_** |
| BN341_15110 | ATP-dependent Clp protease ATP-binding subunit ClpX | protein folding | ATP binding,  zinc ion binding,  protein dimerization activity,  unfolded protein binding | 2.187 | 2.30E-46 | 5.90E-44 |
| BN341_470 | Putative transmembrane transport protein | transmembrane transport | zinc ion binding,  protein dimerization activity | 2.182 | 7.60E-12 | 1.00E-10 |
| BN341_17040 | Urease beta subunit | nitrogen compound metabolic process | urease activity,  nickel cation binding,  hydrolase activity,  acting on carbon-nitrogen (but not peptide) bonds | 2.176 | 9.30E-29 | 8.40E-27 |
| BN341_5540 | hypothetical protein |  |  | 2.176 | 2.40E-08 | 1.90E-07 |
| BN341_4310 | Gamma-glutamyltranspeptidase | glutathione catabolic process | glutathione hydrolase activity | 2.165 | 1.50E-38 | 2.80E-36 |
| BN341_8660 | hypothetical protein |  |  | 2.155 | 1.80E-05 | 9.00E-05 |
| BN341_140 | hypothetical protein |  |  | 2.127 | 2.10E-06 | 1.20E-05 |
| BN341_15280 | Transcription antitermination protein NusG | regulation of transcription,  DNA-templated,  regulation of DNA-templated transcription,  elongation |  | 2.127 | 4.90E-10 | 5.10E-09 |
| BN341_9650 | Fructose-bisphosphate aldolase class II | carbohydrate metabolic process, glycolytic process,  fructose 1,6-bisphosphate metabolic process | catalytic activity,  fructose-bisphosphate aldolase activity, zinc ion binding,  aldehyde-lyase activity | 2.125 | 5.00E-27 | 4.30E-25 |
| BN341_4620 | Molybdenum cofactor biosynthesis protein MoaA | Mo-molybdopterin cofactor biosynthetic process | catalytic activity,  metal ion binding,  iron-sulfur cluster binding,  4 iron, 4 sulfur cluster binding | 2.117 | 1.20E-10 | 1.30E-09 |
| BN341_1640 | disulphide isomerase |  |  | 2.112 | 1.00E-32 | 1.10E-30 |
| BN341_11390 | hypothetical protein |  |  | 2.111 | 4.50E-05 | 2.20E-04 |
| BN341_4260 | LSU ribosomal protein L13p (L13Ae) | translation | structural constituent of ribosome | 2.095 | 1.20E-09 | 1.10E-08 |
| BN341_4150 | Biopolymer transport protein ExbD/TolR | transmembrane transport | transmembrane transporter activity | 2.083 | 1.70E-12 | 2.60E-11 |
| BN341_9870 | hypothetical protein |  |  | 2.082 | 1.20E-04 | 5.10E-04 |
| **Id** | **Description** | **Biological process** | **Molecular function** | **Fold change** | ***p*-value** | **p_adj_** |
| BN341_7530 | SSU ribosomal protein S10p (S20e) | translation | RNA binding,  structural constituent of ribosome | 2.081 | 2.00E-08 | 1.60E-07 |
| BN341_6230 | Phosphopantothenoylcysteine decarboxylase | coenzyme A biosynthetic process, pantothenate catabolic process | catalytic activity,  phosphopantothenate--cysteine ligase activity,  phosphopantothenoylcysteine decarboxylase activity,  FMN binding | 2.080 | 4.90E-08 | 3.70E-07 |
| BN341_14190 | Dihydrofolate synthase | biosynthetic process,  folic acid-containing compound biosynthetic process | tetrahydrofolylpolyglutamate synthase activity,  ATP binding,  ligase activity | 2.067 | 7.80E-07 | 5.00E-06 |
| BN341_14020 | conserved hypothetical protein |  |  | 2.058 | 1.89E-03 | 6.04E-03 |
| BN341_160 | Probable outer membrane component of multidrug efflux pump | transmembrane transport | efflux transmembrane transporter activity | 2.043 | 6.50E-16 | 1.40E-14 |
| BN341_19750 | ATP synthase delta chain | ATP synthesis coupled proton transport | proton-transporting ATP synthase activity,  rotational mechanism | 2.031 | 1.90E-09 | 1.70E-08 |
| BN341_4250 | SSU ribosomal protein S9p (S16e) | translation | structural constituent of ribosome | 2.018 | 2.20E-12 | 3.30E-11 |
| BN341_13030 | putative thioredoxin |  |  | 2.013 | 4.10E-04 | 1.55E-03 |
| BN341_19320 | Homolog of fucose/glucose/galactose permeases |  |  | 2.000 | 5.90E-15 | 1.10E-13 |
| BN341_5240 | Putative membrane protein YeiH |  |  | 2.000 | 6.20E-09 | 5.40E-08 |
