# Supplementary material for: Distinct transcriptome signatures of Helicobacter suis and Helicobacter heilmannii strains upon adherence to human gastric epithelial cells
Source: Vet Res. 2020 May 7;51:62. doi: 10.1186/s13567-020-00786-w (PMC7206758; doi:10.1186/s13567-020-00786-w)
Supplement: Supplementary file 4 — Additional file 4. List of 83 significantly down-regulated H. heilmannii genes in cases compared to controls (with p adj ≤ 0.01; fold change ≤ -2). [file 13567_2020_786_MOESM4_ESM.docx]

| **Id** | **Description** | **Biological process** | **Molecular function** | **Fold change** | ***p*-value** | **p_adj_** |
| --- | --- | --- | --- | --- | --- | --- |
| BN341_11170 | hypothetical protein |  |  | -2.008 | 3.37E-04 | 1.29E-03 |
| BN341_14810 | hypothetical protein |  |  | -2.012 | 3.36E-04 | 1.29E-03 |
| BN341_6750 | Molybdopterin biosynthesis Mog protein, molybdochelatase |  |  | -2.016 | 5.33E-05 | 2.52E-04 |
| BN341_5040 | hypothetical protein |  |  | -2.020 | 1.02E-03 | 3.49E-03 |
| BN341_4040 | C4-dicarboxylate transporter DcuA | C4-dicarboxylate transport | C4-dicarboxylate transmembrane transporter activity | -2.024 | 5.25E-19 | 1.83E-17 |
| BN341_4060 | putative |  | / | -2.033 | 1.44E-18 | 4.58E-17 |
| BN341_18190 | 3-methyl-2-oxobutanoate hydroxymethyltransferase | pantothenate biosynthetic process | catalytic activity, 3-methyl-2-oxobutanoate hydroxymethyltransferase activity | -2.037 | 2.18E-04 | 8.79E-04 |
| BN341_12060 | KH domain RNA binding protein YlqC |  |  | -2.045 | 2.90E-03 | 8.83E-03 |
| BN341_13680 | CDP-diacylglycerol-serine O-phosphatidyltransferase | phospholipid biosynthetic process | phosphotransferase activity, for other substituted phosphate groups | -2.066 | 2.91E-15 | 5.60E-14 |
| BN341_4810 | hypothetical protein |  |  | -2.066 | 1.17E-04 | 5.09E-04 |
| BN341_2070 | NADH dehydrogenase | oxidation-reduction process | oxidoreductase activity | -2.070 | 4.95E-04 | 1.82E-03 |
| BN341_6440 | putative protease (EC:3.4.-) |  |  | -2.070 | 6.33E-16 | 1.33E-14 |
| BN341_18660 | hypothetical protein |  |  | -2.079 | 9.69E-23 | 5.01E-21 |
| BN341_890 | TrkA | potassium ion transport | cation transmembrane transporter activity | -2.092 | 5.84E-04 | 2.09E-03 |
| BN341_4820 | Formate dehydrogenase-O, major subunit | oxidation-reduction process | oxidoreductase activity | -2.096 | 4.55E-18 | 1.28E-16 |
| BN341_12340 | hypothetical protein |  |  | -2.105 | 2.34E-16 | 5.17E-15 |
| BN341_3090 | outer membrane protein 13 |  |  | -2.105 | 2.09E-14 | 3.70E-13 |
| BN341_4450 | 2-oxoglutarate oxidoreductase, delta subunit, putative |  |  | -2.110 | 2.37E-03 | 7.41E-03 |
| BN341_16430 | Quinone-reactive Ni/Fe-hydrogenase small chain precursor | oxidation-reduction process | ferredoxin hydrogenase activity, iron-sulfur cluster binding | -2.114 | 2.97E-19 | 1.05E-17 |
| BN341_9620 | Protein crcB homolog |  | peptidyl-prolyl *cis,trans*-isomerase activity | -2.114 | 1.57E-05 | 8.03E-05 |
| **Id** | **Description** | **Biological process** | **Molecular function** | **Fold change** | ***p*-value** | **p_adj_** |
| BN341_4690 | hypothetical protein (EC:3.1.21.4) |  |  | -2.141 | 1.41E-18 | 4.56E-17 |
| BN341_12690 | Polyferredoxin NapH (periplasmic nitrate reductase) |  |  | -2.155 | 4.52E-18 | 1.28E-16 |
| BN341_3170 | 8-amino-7-oxononanoate synthase | metabolic process,  biosynthetic process | catalytic activity,  transferase activity,  pyridoxal phosphate binding | -2.160 | 2.98E-13 | 4.85E-12 |
| BN341_17150 | putative type II DNA modification enzyme (methyltransferase) | DNA methylation,  N-4 methylation of cytosine | DNA binding,  N-methyltransferase activity,  site-specific DNA-methyltransferase (cytosine-N4-specific) activity | -2.170 | 1.50E-05 | 7.70E-05 |
| BN341_3320 | hypothetical protein |  |  | -2.179 | 1.35E-36 | 2.03E-34 |
| BN341_4170 | Integral membrane protein |  |  | -2.183 | 4.25E-22 | 2.14E-20 |
| BN341_9940 | hypothetical protein |  |  | -2.188 | 6.73E-39 | 1.52E-36 |
| BN341_13230 | Acyl-phosphate:glycerol-3-phosphate O-acyltransferase PlsY | phospholipid biosynthetic process | acyl-phosphate glycerol-3-phosphate acyltransferase activity | -2.208 | 8.78E-08 | 6.35E-07 |
| BN341_260 | hypothetical protein |  |  | -2.217 | 2.33E-10 | 2.53E-09 |
| BN341_6380 | outer membrane protein 27 |  |  | -2.217 | 6.81E-17 | 1.69E-15 |
| BN341_17500 | Prolipoprotein diacylglyceryl transferase | lipoprotein biosynthetic process | transferase activity, transferring glycosyl groups | -2.222 | 1.21E-04 | 5.27E-04 |
| BN341_18910 | Holo-[acyl-carrier protein] synthase | fatty acid biosynthetic process | magnesium ion binding,  holo-[acyl-carrier-protein] synthase activity | -2.222 | 1.53E-14 | 2.74E-13 |
| BN341_16270 | Phosphoserine phosphatase |  |  | -2.227 | 1.23E-34 | 1.71E-32 |
| BN341_11500 | Rna-binding protein |  | nucleic acid binding | -2.232 | 1.30E-16 | 3.05E-15 |
| BN341_12930 | Cytochrome C553 (soluble cytochrome f) |  | electron transfer activity,  heme binding | -2.252 | 8.83E-11 | 1.02E-09 |
| BN341_16320 | Aspartyl-tRNA(Asn) amidotransferase subunit C | regulation of translational fidelity |  | -2.262 | 1.49E-18 | 4.64E-17 |
| BN341_4570 | [NiFe] hydrogenase metallocenter assembly protein HypD |  | metal ion binding | -2.273 | 5.41E-23 | 2.88E-21 |
| BN341_7960 | hypothetical protein |  |  | -2.273 | 1.09E-12 | 1.72E-11 |
| BN341_15670 | GTP-binding and nucleic acid-binding protein YchF |  | GTP binding | -2.278 | 4.49E-20 | 1.77E-18 |
| **Id** | **Description** | **Biological process** | **Molecular function** | **Fold change** | ***p*-value** | **p_adj_** |
| BN341_17990 | 6,7-dimethyl-8-ribityllumazine synthase | riboflavin biosynthetic process | 6,7-dimethyl-8-ribityllumazine synthase activity | -2.278 | 2.06E-10 | 2.26E-09 |
| BN341_2080 | Threonine dehydrogenase and related Zn-dependent dehydrogenases | oxidation-reduction process | zinc ion binding,  oxidoreductase activity | -2.278 | 4.94E-07 | 3.25E-06 |
| BN341_180 | DNA-binding protein HU |  | DNA binding | -2.294 | 5.70E-06 | 3.19E-05 |
| BN341_4480 | hypothetical protein |  |  | -2.299 | 1.04E-04 | 4.65E-04 |
| BN341_18110 | hypothetical protein |  |  | -2.304 | 4.15E-24 | 2.42E-22 |
| BN341_6740 | Molybdenum cofactor biosynthesis protein MoaC | Mo-molybdopterin cofactor biosynthetic process |  | -2.331 | 1.69E-04 | 7.01E-04 |
| BN341_9360 | 4-diphosphocytidyl-2-C-methyl-D-erythritol kinase | terpenoid biosynthetic process | ATP binding,  4-(cytidine 5'-diphospho)-2-C-methyl-D-erythritol kinase activity | -2.336 | 2.11E-09 | 1.92E-08 |
| BN341_6370 | hypothetical protein |  |  | -2.347 | 1.48E-16 | 3.40E-15 |
| BN341_18090 | RecA protein | DNA metabolic process, DNA repair | DNA binding,  single-stranded DNA binding,  ATP binding,  DNA-dependent ATPase activity | -2.358 | 8.64E-16 | 1.78E-14 |
| BN341_6140 | UDP-N-acetylglucosamine-N-acetylmuramyl-(pentapeptide) pyrophosphoryl-undecaprenol N-acetylglucosamine transferase | carbohydrate metabolic process, lipid glycosylation | transferase activity,  transferring hexosyl groups, undecaprenyldiphospho-muramoylpentapeptide beta-N-acetylglucosaminyl-transferase activity | -2.358 | 3.71E-23 | 2.04E-21 |
| BN341_16510 | hypothetical protein |  |  | -2.404 | 3.41E-04 | 1.29E-03 |
| BN341_18480 | unknown | DNA modification | DNA binding | -2.427 | 1.32E-11 | 1.73E-10 |
| BN341_2750 | hypothetical protein |  |  | -2.451 | 6.08E-05 | 2.83E-04 |
| BN341_510 | outer membrane protein (omp4) |  |  | -2.463 | 1.69E-07 | 1.18E-06 |
| BN341_4940 | putative |  |  | -2.475 | 5.73E-29 | 5.46E-27 |
| BN341_1710 | hypothetical protein |  |  | -2.494 | 2.17E-04 | 8.79E-04 |
| BN341_6100 | Heat shock protein GrpE | protein folding | adenyl-nucleotide exchange factor activity,  protein homodimerization activity, chaperone binding | -2.494 | 7.03E-09 | 6.03E-08 |
| **Id** | **Description** | **Biological process** | **Molecular function** | **Fold change** | ***p*-value** | **p_adj_** |
| BN341_13080 | hypothetical protein |  |  | -2.519 | 5.24E-07 | 3.42E-06 |
| BN341_2970 | Tellurium resistance protein | response to stress |  | -2.519 | 9.30E-26 | 7.01E-24 |
| BN341_4220 | hypothetical protein |  |  | -2.519 | 9.74E-12 | 1.31E-10 |
| BN341_9340 | Peptidyl-prolyl *cis,trans*-isomerase | protein peptidyl-prolyl isomerization,  protein folding | peptidyl-prolyl *cis,trans*-isomerase activity | -2.532 | 2.18E-11 | 2.74E-10 |
| BN341_18000 | Transcription termination protein NusB | DNA-templated transcription, termination,  regulation of transcription,  DNA-templated | RNA binding | -2.545 | 2.85E-14 | 5.00E-13 |
| BN341_3310 | hypothetical protein |  |  | -2.558 | 1.40E-20 | 5.88E-19 |
| BN341_1560 | Outer membrane protein |  |  | -2.584 | 4.90E-37 | 8.05E-35 |
| BN341_12070 | SSU ribosomal protein S16p | translation | structural constituent of ribosome | -2.604 | 3.58E-06 | 2.07E-05 |
| BN341_220 | hypothetical protein |  |  | -2.710 | 2.58E-05 | 1.28E-04 |
| BN341_9370 | tmRNA-binding protein SmpB |  | RNA binding | -2.725 | 8.59E-09 | 7.23E-08 |
| BN341_13120 | hypothetical protein |  |  | -2.732 | 2.95E-09 | 2.62E-08 |
| BN341_9350 | Carbon storage regulator | regulation of carbohydrate metabolic process,  mRNA catabolic process | RNA binding | -2.747 | 2.90E-07 | 2.00E-06 |
| BN341_6910 | Modification methylase |  | methyltransferase activity | -2.778 | 4.50E-07 | 3.03E-06 |
| BN341_5350 | hypothetical protein |  |  | -2.841 | 3.54E-17 | 9.15E-16 |
| BN341_5900 | hypothetical protein |  |  | -2.857 | 1.77E-05 | 8.92E-05 |
| BN341_10590 | hypothetical protein |  |  | -2.865 | 5.07E-09 | 4.41E-08 |
| BN341_16400 | Hydrogenase maturation protease |  | enzyme activator activity,  peptidase activity | -3.012 | 5.42E-15 | 1.01E-13 |
| BN341_6150 | Flagellar assembly factor FliW | bacterial-type flagellum assembly |  | -3.012 | 9.74E-20 | 3.75E-18 |
| BN341_2960 | Tellurium resistance protein TerD | response to stress |  | -3.115 | 1.08E-16 | 2.59E-15 |
| BN341_10030 | dicarboxylic acid transporter PcaT | transmembrane transport | transporter activity | -3.125 | 1.30E-48 | 3.91E-46 |
| BN341_5960 | hypothetical protein |  |  | -3.155 | 2.21E-21 | 1.05E-19 |
| **Id** | **Description** | **Biological process** | **Molecular function** | **Fold change** | ***p*-value** | **p_adj_** |
| BN341_17260 | Mn2+/Fe2+ transporter, NRAMP family | metal ion transport | metal ion transmembrane transporter activity | -3.215 | 1.40E-06 | 8.53E-06 |
| BN341_7680 | hypothetical protein |  |  | -3.289 | 6.80E-123 | 1.20E-119 |
| BN341_410 | hypothetical protein |  |  | -3.584 | 2.67E-60 | 1.61E-57 |
| BN341_250 | hypothetical protein |  |  | -3.922 | 3.10E-08 | 2.42E-07 |
| BN341_5740 | SSU ribosomal protein S12p (S23e) | translation | structural constituent of ribosome | -5.181 | 3.28E-12 | 4.83E-11 |
| BN341_3450 | outer membrane protein (omp30) |  |  | -5.348 | 7.04E-33 | 8.49E-31 |
