# Supplementary figures and images for: Distinct transcriptome signatures of Helicobacter suis and Helicobacter heilmannii strains upon adherence to human gastric epithelial cells
Source: Vet Res. 2020 May 7;51:62. doi: 10.1186/s13567-020-00786-w (PMC7206758; doi:10.1186/s13567-020-00786-w)

A

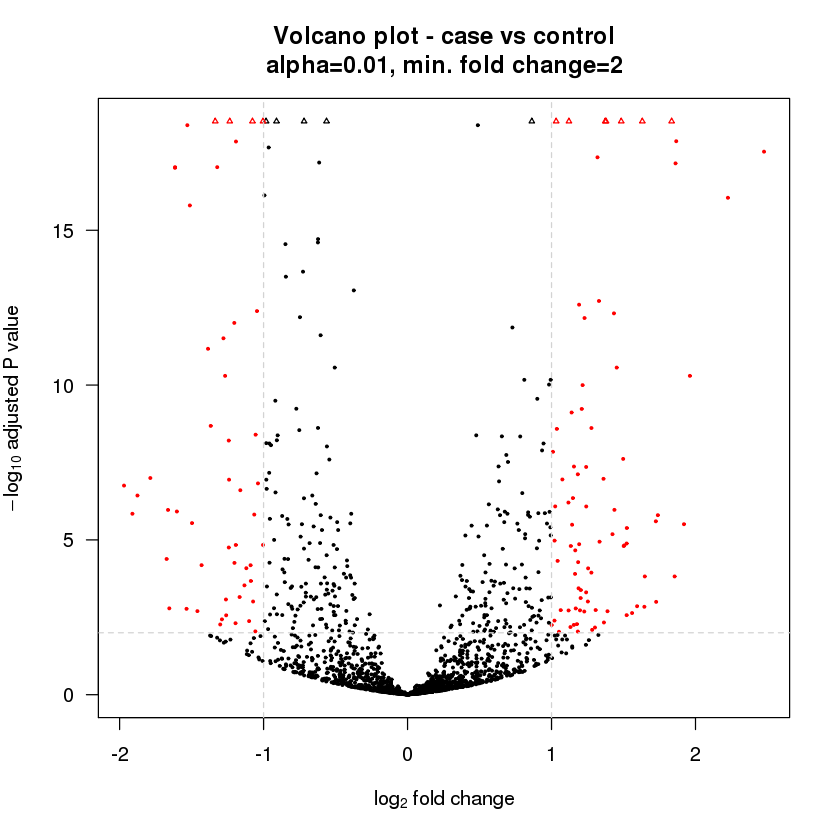


B

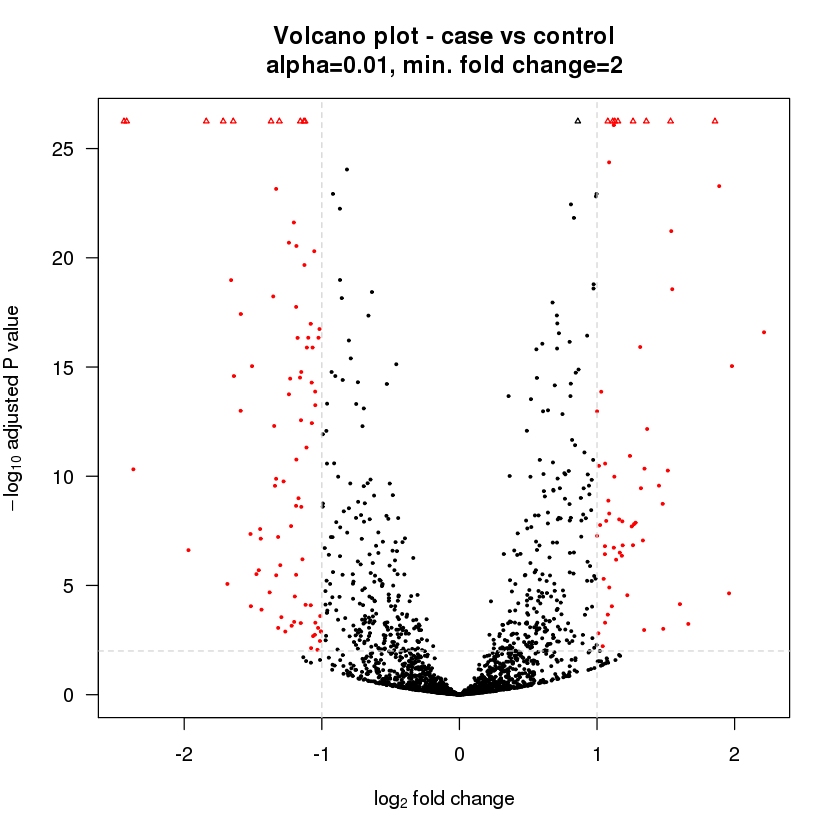

Supplement: Supplementary file 5 — Additional file 5. Volcano plots of the comparisons (case versus control). Red dots represent significantly differentially expressed genes (padj ≤ 0.01, fold change ≥ 2) in MKN7-bound helicobacters (i.e. cases) compared to unbound helicobacters (i.e. controls). (A) Volcano plot of the comparison (case versus control) for H. suis. (B) Volcano plot of the comparison (case versus control) for H. heilmannii. [file 13567_2020_786_MOESM5_ESM.docx]

A B


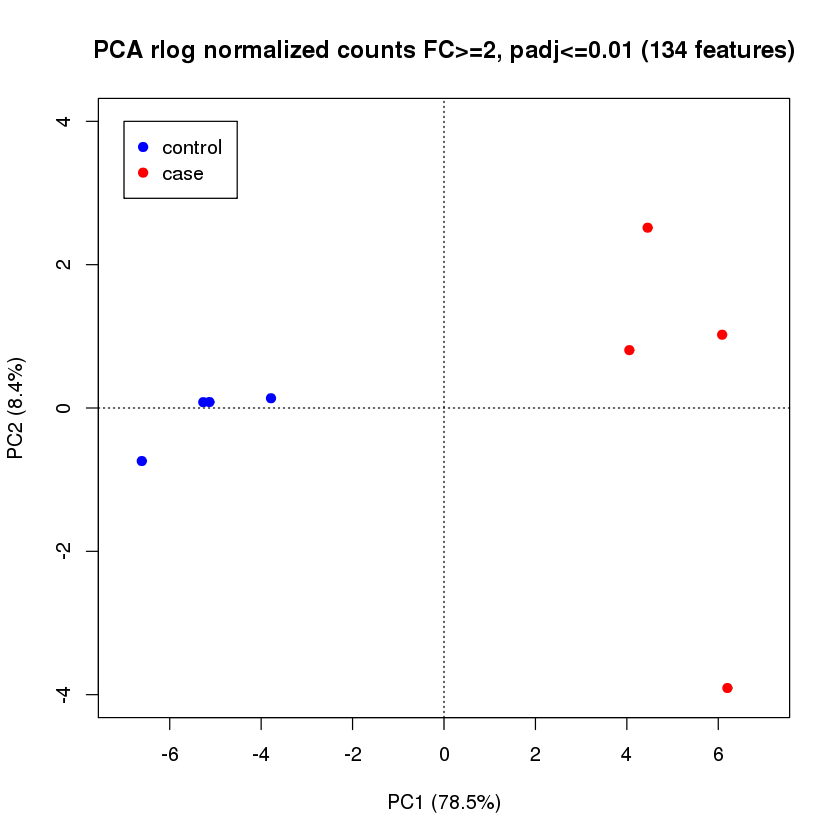

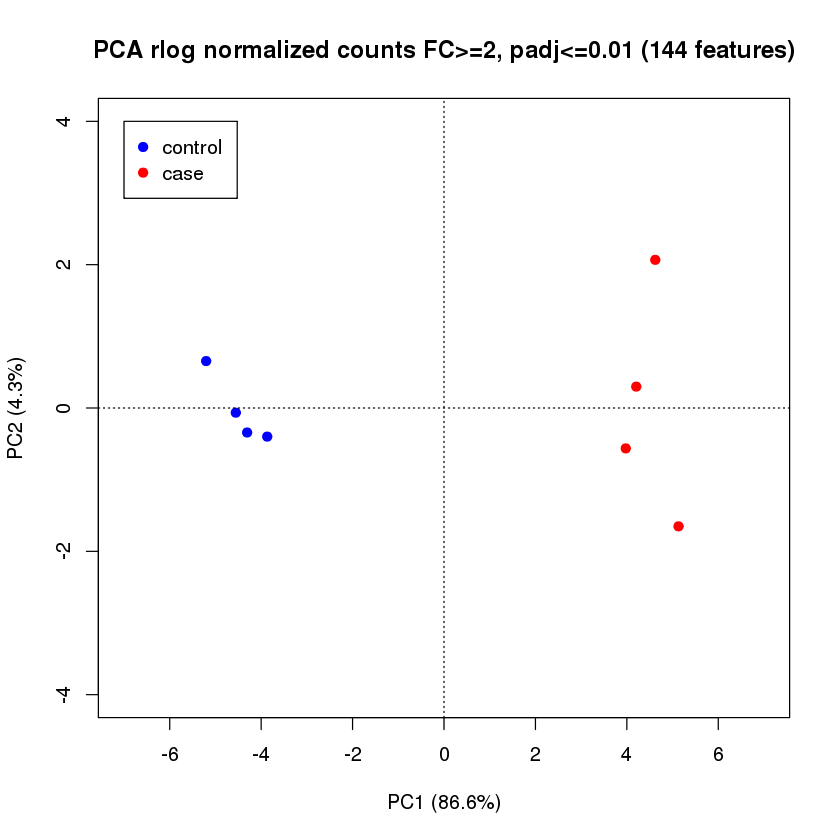

Supplement: Supplementary file 6 — Additional file 6. Plots of the principal component analysis (PCA) of the restricted set of differentially expressed features. First 2 components (PC1 and PC2) of a PCA, with percentages of variance associated with each axis. These plots show the separation between samples based on the main sources of variation found in the rlog-transformed data sets (padj ≤ 0.01, fold change ≥ 2). (A) PCA-plot for H. suis (134 differentially expressed features) (B) PCA-plot for H. heilmannii (143 differentially expressed features). Blue dots indicate control samples (i.e. unbound Helicobacter); red dots indicate cases (i.e. Helicobacter bound to MKN7 cells). In both PCA plots, the sample groups (i.e. case and control) are well separated, indicating that the main variability within each experiment came from biological differences between cases and controls. [file 13567_2020_786_MOESM6_ESM.docx]
