# Supplementary material for: Distinct transcriptome signatures of Helicobacter suis and Helicobacter heilmannii strains upon adherence to human gastric epithelial cells
Source: Vet Res. 2020 May 7;51:62. doi: 10.1186/s13567-020-00786-w (PMC7206758; doi:10.1186/s13567-020-00786-w)
Supplement: Supplementary file 7 — Additional file 7. H. suis up-regulated genes with (72) and without (11) H. heilmannii homologs according to BLASTp. [file 13567_2020_786_MOESM7_ESM.docx]

| ***H. suis* genID** | **Description** | ***H. heilmannii* genID** | **Description** | **E-value** | **Gaps** | **% identity** | **% positive match** |
| --- | --- | --- | --- | --- | --- | --- | --- |
| 104628.16_00004 | Ferrochelatase | BN341_19560 | Ferrochelatase, protoheme ferro-lyase | 8.77E-166 | 0 | 70.42 | 84.24 |
| 104628.16_00037 | hypothetical protein | BN341_3420 | hypothetical protein | 3.72E-65 | 0 | 57.06 | 74.01 |
| 104628.16_00123 | hypothetical protein | BN341_14820 | NADH-ubiquinone oxidoreductase subunit | 1.49E-111 | 9 | 53.93 | 69.38 |
| 104628.16_00145 | 3-phosphoshikimate 1-carboxyvinyltransferase | BN341_11770 | 5-enolpyruvylshikimate-3-phosphate synthase | 0 | 1 | 66.74 | 78.14 |
| 104628.16_00155 | Fumarate reductase cytochrome b subunit | BN341_10000 | Fumarate reductase cytochrome b subunit | 4.88E-149 | 1 | 85.37 | 90.91 |
| 104628.16_00165 | hypothetical protein | BN341_10130 | hypothetical protein | 5.36E-80 | 0 | 75.00 | 85.42 |
| 104628.16_00173 | Mechanosensitive ion channel | BN341_2370 | Putative mechanosensitive ion channel | 0 | 5 | 73.68 | 85.71 |
| 104628.16_00174 | tumor necrosis factor alpha-inducing protein | BN341_12720 | putative | 1.99E-86 | 4 | 62.90 | 80.11 |
| 104628.16_00201 | hypothetical protein | BN341_15080 | hypothetical protein | 5.2E-110 | 7 | 59.36 | 74.20 |
| 104628.16_00302 | Oligopeptide-binding protein AppA precursor | BN341_19310 | Putative peptide ABC-transport system periplasmic peptide-binding protein | 0 | 7 | 74.60 | 85.08 |
| 104628.16_00303 | Nickel transport system permease protein NikB | BN341_19300 | peptide ABC transporter, permease protein | 4.19E-149 | 1 | 66.77 | 82.11 |
| 104628.16_00341 | hypothetical protein | BN341_2600 | hypothetical protein | 7.29E-08 | 10 | 30.25 | 55.46 |
| 104628.16_00348 | ribonuclease BN/unknown domain fusion protein | BN341_18010 | Ribonuclease BN | 8.23E-158 | 0 | 77.37 | 87.23 |
| 104628.16_00362 | hypothetical protein | BN341_9490 | hypothetical protein | 1.61E-69 | 0 | 78.15 | 88.24 |
| 104628.16_00393 | Urease accessory protein UreH | BN341_17120 | Urease accessory protein UreD | 4.27E-139 | 1 | 69.15 | 82.53 |
| 104628.16_00395 | Urease accessory protein UreF | BN341_17100 | Urease accessory protein UreF | 1.52E-137 | 0 | 80.09 | 88.74 |
| 104628.16_00438 | DNA primase | BN341_8740 | DNA primase | 2.67E-41 | 12 | 32.84 | 52.99 |
| 104628.16_00443 | hypothetical protein | BN341_5270 | FIG00710779: hypothetical protein | 5.89E-65 | 0 | 84.26 | 91.67 |
| 104628.16_00450 | Ribosomal protein S12 methylthiotransferase RimO | BN341_17780 | Ribosomal protein S12p Asp88 (*E. coli*) methylthiotransferase | 0 | 0 | 74.01 | 87.01 |
| 104628.16_00453 | hypothetical protein | BN341_17750 | hypothetical protein | 7.71E-32 | 8 | 42.76 | 59.87 |
| 104628.16_00465 | hypothetical protein | BN341_4800 | Putative formate dehydrogenase-specific chaperone | 1.61E-102 | 0 | 68.50 | 82.50 |
| 104628.16_00480 | hypothetical protein | BN341_7760 | hypothetical protein | 1.44E-48 | 27 | 48.02 | 62.70 |
| 104628.16_00561 | hypothetical protein | BN341_8340 | hypothetical protein | 1.72E-12 | 1 | 63.04 | 73.91 |
| 104628.16_00573 | hypothetical protein | BN341_10650 | hypothetical protein | 9.65E-77 | 0 | 73.57 | 87.86 |
| 104628.16_00648 | hypothetical protein | BN341_6130 | FIG00711213: hypothetical protein | 3.17E-46 | 1 | 82.02 | 86.52 |
| ***H. suis* genID** | **Description** | ***H. heilmannii* genID** | **Description** | **E-value** | **Gaps** | **% identity** | **% positive match** |
| 104628.16_00717 | hypothetical protein | BN341_18380 | hypothetical protein | 1.92E-23 | 0 | 72.73 | 83.64 |
| 104628.16_00759 | Putative nickel-responsive regulator | BN341_17510 | Nickel responsive regulator NikR | 1.21E-92 | 2 | 86.58 | 93.29 |
| 104628.16_00761 | Ribosomal large subunit pseudouridine synthase D | BN341_17490 | ribosomal large subunit pseudouridine synthase C(EC:4.2.1.70) | 2.8E-102 | 1 | 59.20 | 75.60 |
| 104628.16_00764 | GTP cyclohydrolase 1 type 2 | BN341_17460 | FIG137478: Hypothetical protein | 5.89E-119 | 0 | 62.15 | 78.88 |
| 104628.16_00789 | Farnesyl diphosphate synthase | BN341_16060 | Octaprenyl-diphosphate synthase | 4.14E-145 | 0 | 67.57 | 80.07 |
| 104628.16_00791 | hypothetical protein | BN341_16080 | hypothetical protein | 8.81E-129 | 0 | 78.10 | 90.00 |
| 104628.16_00811 | hypothetical protein | BN341_9120 | conserved hypothetical protein | 2.49E-46 | 1 | 55.12 | 70.87 |
| 104628.16_00837 | hypothetical protein | BN341_2730 | hypothetical protein | 8.61E-47 | 49 | 34.28 | 50.26 |
| 104628.16_00843 | Amidophosphoribosyltransferase precursor | BN341_12460 | Amidophosphoribosyltransferase | 4.93E-116 | 7 | 64.21 | 75.65 |
| 104628.16_00868 | preprotein translocase subunit SecF | BN341_14220 | Protein-export membrane protein SecF (TC 3.A.5.1.1) | 5.67E-170 | 1 | 80.06 | 90.34 |
| 104628.16_00999 | hypothetical protein | BN341_10980 | Menaquinone via futalosine step 1 | 5.09E-126 | 0 | 72.17 | 84.35 |
| 104628.16_01010 | Acyl-CoA thioester hydrolase YbgC | BN341_11120 | 4-hydroxybenzoyl-CoA thioesterase family active site | 1.38E-58 | 3 | 65.35 | 79.53 |
| 104628.16_01022 | ABC-2 family transporter protein | BN341_9690 | ABC-type multidrug transport system, permease component | 9.38E-157 | 0 | 66.56 | 82.04 |
| 104628.16_01030 | Transaldolase | BN341_9800 | Transaldolase | 4.83E-139 | 25 | 63.33 | 73.64 |
| 104628.16_01061 | Methyl-accepting chemotaxis protein PctC | BN341_2410 | hypothetical protein | 2.04E-23 | 13 | 28.85 | 48.62 |
| 104628.16_01096 | flagellar basal body P-ring biosynthesis protein FlgA | BN341_11900 | flagellar basal body P-ring biosynthesis protein | 8.69E-102 | 6 | 69.57 | 84.54 |
| 104628.16_01099 | Thymidylate kinase | BN341_11880 | Thymidylate kinase | 7.09E-85 | 2 | 64.77 | 81.87 |
| 104628.16_01120 | PD-(D/E)XK nuclease superfamily protein | BN341_6610 | hypothetical protein | 0 | 14 | 54.81 | 71.30 |
| 104628.16_01165 | putative copper-transporting ATPase PacS | BN341_11450 | Lead, cadmium, zinc and mercury transporting ATPase | 0 | 6 | 64.02 | 78.91 |
| 104628.16_01166 | tRNA1(Val) (adenine(37)-N6)-methyltransferase | BN341_11440 | tRNA (adenine37-N(6))-methyltransferase TrmN6 | 7.95E-125 | 1 | 70.09 | 85.90 |
| 104628.16_01189 | Octaprenyl-diphosphate synthase | BN341_840 | Octaprenyl-diphosphate synthase | 5.72E-147 | 0 | 67.68 | 79.80 |
| 104628.16_01201 | Putative phosphoribosyl transferase/MT0597 | BN341_13350 | FIG00387830: hypothetical protein | 1.17E-135 | 0 | 83.11 | 91.78 |
| 104628.16_01211 | hypothetical protein | BN341_10530 | hypothetical protein | 9.53E-107 | 0 | 88.44 | 95.95 |
| 104628.16_01253 | 3-deoxy-manno-octulosonate cytidylyltransferase | BN341_13040 | 3-deoxy-manno-octulosonate cytidylyltransferase | 1.99E-135 | 0 | 77.92 | 88.74 |
| 104628.16_01297 | Phosphorylase superfamily protein | BN341_1660 | purine nucleoside phosphorylase (punB) | 2.09E-77 | 2 | 63.01 | 78.03 |
| 104628.16_01310 | Soluble lytic murein transglycosylase precursor | BN341_19020 | Soluble lytic murein transglycosylase precursor | 0 | 5 | 61.65 | 78.17 |
| 104628.16_01324 | D-glycero-alpha-D-manno-heptose-1,7-bisphosphate 7-phosphatase | BN341_19150 | D-glycero-D-manno-heptose 1,7-bisphosphate phosphatase | 1.93E-77 | 3 | 64.91 | 78.95 |
| ***H. suis* genID** | **Description** | ***H. heilmannii* genID** | **Description** | **E-value** | **Gaps** | **% identity** | **% positive match** |
| 104628.16_01335 | hypothetical protein | BN341_4510 | hypothetical protein | 8.44E-115 | 22 | 60.87 | 74.64 |
| 104628.16_01347 | hypothetical protein | BN341_4390 | hypothetical protein | 2.07E-28 | 4 | 40.95 | 53.54 |
| 104628.16_01363 | Glycosyltransferase family 9 (heptosyltransferase) | BN341_14140 | ADP-heptose--lipooligosaccharide heptosyltransferase II | 0.000247 | 4 | 24.18 | 47.25 |
| 104628.16_01371 | Methyl-accepting chemotaxis protein 4 | BN341_11610 | methyl-accepting chemotaxis protein | 0 | 2 | 62.48 | 80.38 |
| 104628.16_01377 | 30S ribosomal protein S20 | BN341_2310 | SSU ribosomal protein S20p | 2.03E-52 | 0 | 86.81 | 94.51 |
| 104628.16_01380 | hypothetical protein | BN341_2270 | hypothetical protein | 1.38E-61 | 0 | 58.55 | 72.37 |
| 104628.16_01404 | putative inorganic polyphosphate/ATP-NAD kinase | BN341_6940 | NAD kinase | 4.84E-118 | 0 | 64.48 | 76.83 |
| 104628.16_01486 | NAD(P)H-quinone oxidoreductase chain 4 1 | BN341_10360 | NADH-ubiquinone oxidoreductase chain M | 0 | 13 | 73.20 | 84.60 |
| 104628.16_01489 | NADH-quinone oxidoreductase subunit J | BN341_10330 | NADH-ubiquinone oxidoreductase chain J | 3.46E-67 | 4 | 59.54 | 76.30 |
| 104628.16_01497 | NAD(P)H-quinone oxidoreductase subunit 3 | BN341_10250 | NADH ubiquinone oxidoreductase chain A | 1.08E-74 | 0 | 82.11 | 93.50 |
| 104628.16_01504 | hypothetical protein | BN341_19480 | hypothetical protein | 5.44E-67 | 0 | 69.40 | 87.31 |
| 104628.16_01508 | hypothetical protein | BN341_8480 | hypothetical protein | 9.47E-19 | 1 | 51.47 | 72.06 |
| 104628.16_01509 | hypothetical protein | BN341_2730 | hypothetical protein | 2.56E-25 | 14 | 43.31 | 60.63 |
| 104628.16_01539 | CDP-diacylglycerol--glycerol-3-phosphate 3-phosphatidyltransferase | BN341_13830 | CDP-diacylglycerol--glycerol-3-phosphate 3-phosphatidyltransferase | 9.31E-79 | 0 | 68.82 | 79.57 |
| 104628.16_01557 | L-seryl-tRNA(Sec) selenium transferase | BN341_4990 | L-seryl-tRNA(Sec) selenium transferase | 0 | 7 | 63.66 | 78.01 |
| 104628.16_01560 | putative undecaprenyl-phosphate N-acetylglucosaminyl 1-phosphate transferase | BN341_4920 | Undecaprenyl-phosphate N-acetylglucosaminyl 1-phosphate transferase | 3.32E-127 | 0 | 62.14 | 77.02 |
| 104628.16_01569 | paraquat-inducible protein B | BN341_5580 | ABC transporter, periplasmic substrate-binding protein, putative | 1.24E-154 | 7 | 72.47 | 85.02 |
| 104628.16_01571 | putative phospholipid ABC transporter permease protein MlaE | BN341_5600 | ABC-type transport system involved in resistance to organic solvents, permease component | 0 | 0 | 85.25 | 95.63 |
| 104628.16_01608 | hypothetical protein | BN341_8410 | hypothetical protein | 2.56E-50 | 0 | 57.14 | 72.27 |
| 104628.16_01614 | hypothetical protein | BN341_9940 | hypothetical protein | 4.92E-34 | 0 | 52.94 | 78.82 |
| 104628.16_00432 | hypothetical protein |  |  |  |  |  |  |
| 104628.16_00482 | putative oxidoreductase YdgJ |  |  |  |  |  |  |
| 104628.16_00558 | hypothetical protein |  |  |  |  |  |  |
| 104628.16_00906 | hypothetical protein |  |  |  |  |  |  |
| 104628.16_01171 | hypothetical protein |  |  |  |  |  |  |
| 104628.16_01370 | CobQ/CobB/MinD/ParA nucleotide binding domain protein |  |  |  |  |  |  |
| ***H. suis* genID** | **Description** | ***H. heilmannii* genID** | **Description** | **E-value** | **Gaps** | **% identity** | **% positive match** |
| 104628.16_01410 | DNA methylase |  |  |  |  |  |  |
| 104628.16_01437 | hypothetical protein |  |  |  |  |  |  |
| 104628.16_01441 | Helix-turn-helix domain protein |  |  |  |  |  |  |
| 104628.16_01470 | hypothetical protein |  |  |  |  |  |  |
| 104628.16_01546 | Fic/DOC family protein |  |  |  |  |  |  |
