# Supplementary material for: Distinct transcriptome signatures of Helicobacter suis and Helicobacter heilmannii strains upon adherence to human gastric epithelial cells
Source: Vet Res. 2020 May 7;51:62. doi: 10.1186/s13567-020-00786-w (PMC7206758; doi:10.1186/s13567-020-00786-w)
Supplement: Supplementary file 8 — Additional file 8. H. suis down-regulated genes with (45) and without (6) H. heilmannii homologs according to BLASTp. [file 13567_2020_786_MOESM8_ESM.docx]

| ***H. suis* genID** | **Description** | ***H. heilmannii* genID** | **Description** | **E-value** | **Gaps** | **% identity** | **% positive match** |
| --- | --- | --- | --- | --- | --- | --- | --- |
| 104628.16_00008 | Glutamate racemase | BN341_19520 | Glutamate racemase | 1.74E-133 | 1 | 72.40 | 82.80 |
| 104628.16_00032 | hypothetical protein | BN341_3570 | hypothetical protein | 8.71E-39 | 5 | 59.65 | 68.42 |
| 104628.16_00033 | hypothetical protein | BN341_3570 | hypothetical protein | 3.79E-51 | 16 | 68.85 | 70.49 |
| 104628.16_00081 | Modification methylase DpnIIB | BN341_4640 | DNA modification methyltransferase | 1.5E-16 | 36 | 26.42 | 39.62 |
| 104628.16_00125 | hypothetical protein | BN341_1520 | Putative periplasmic protein | 5.98E-179 | 2 | 72.49 | 84.24 |
| 104628.16_00154 | Fumarate reductase flavoprotein subunit | BN341_9990 | Succinate dehydrogenase flavoprotein subunit | 0 | 23 | 84.99 | 90.68 |
| 104628.16_00204 | Rod shape-determining protein MreB | BN341_15100 | Rod shape-determining protein MreB | 0 | 0 | 97.10 | 99.71 |
| 104628.16_00222 | 50S ribosomal protein L1 | BN341_15300 | LSU ribosomal protein L1p (L10Ae) | 2E-154 | 0 | 87.98 | 97.00 |
| 104628.16_00223 | 50S ribosomal protein L10 | BN341_15310 | LSU ribosomal protein L10p (P0) | 8.37E-95 | 0 | 78.75 | 91.25 |
| 104628.16_00224 | 50S ribosomal protein L7/L12 | BN341_15320 | LSU ribosomal protein L7/L12 (P1/P2) | 4.58E-60 | 0 | 91.94 | 94.35 |
| 104628.16_00269 | UDP-2-acetamido-3-amino-2,3-dideoxy-D-glucuronate N-acetyltransferase | BN341_12130 | hypothetical protein | 3.12E-11 | 6 | 31.67 | 49.17 |
| 104628.16_00282 | Acyl carrier protein | BN341_6510 | Acyl carrier protein | 7.13E-50 | 0 | 97.44 | 100 |
| 104628.16_00283 | 3-oxoacyl-[acyl-carrier-protein] synthase 2 | BN341_6530 | 3-oxoacyl-[acyl-carrier-protein] synthase, KASII | 1.22E-58 | 0 | 84.00 | 93.00 |
| 104628.16_00313 | Dipeptide transport system permease protein DppC | BN341_6660 | Dipeptide transport system permease protein DppC (TC 3.A.1.5.2) | 7.17E-164 | 0 | 79.93 | 90.14 |
| 104628.16_00356 | recombinase A | BN341_18090 | RecA protein | 0 | 1 | 87.65 | 93.82 |
| 104628.16_00398 | Urease subunit beta | BN341_17040 | Urease beta subunit | 0 | 0 | 95.78 | 98.24 |
| 104628.16_00424 | hypothetical protein | BN341_16780 | Acetophenone carboxylase subunit Apc1 | 2.86E-102 | 0 | 71.50 | 84.50 |
| 104628.16_00429 | Acetophenone carboxylase delta subunit | BN341_16750 | Acetophenone carboxylase subunit Apc4 | 0 | 1 | 94.15 | 96.03 |
| 104628.16_00458 | hypothetical protein | BN341_12060 | KH domain RNA binding protein YlqC | 7.35E-42 | 1 | 67.78 | 84.44 |
| 104628.16_00498 | 30S ribosomal protein S10 | BN341_7530 | SSU ribosomal protein S10p (S20e) | 5.65E-71 | 0 | 97.12 | 99.04 |
| 104628.16_00499 | 50S ribosomal protein L3 | BN341_7520 | LSU ribosomal protein L3p (L3e) | 4.09E-31 | 0 | 90.74 | 98.15 |
| 104628.16_00500 | 50S ribosomal protein L4 | BN341_7510 | LSU ribosomal protein L4p (L1e) | 4.36E-67 | 0 | 75.78 | 86.72 |
| 104628.16_00505 | 30S ribosomal protein S3 | BN341_7460 | SSU ribosomal protein S3p (S3e) | 3.78E-156 | 4 | 90.60 | 96.58 |
| 104628.16_00506 | 50S ribosomal protein L16 | BN341_7450 | LSU ribosomal protein L16p (L10e) | 2.02E-97 | 0 | 93.62 | 97.16 |
| 104628.16_00507 | 50S ribosomal protein L29 | BN341_7440 | LSU ribosomal protein L29p (L35e) | 4.96E-27 | 0 | 75.41 | 88.52 |
| ***H. suis* genID** | **Description** | ***H. heilmannii* genID** | **Description** | **E-value** | **Gaps** | **% identity** | **% positive match** |
| 104628.16_00509 | 50S ribosomal protein L14 | BN341_7420 | LSU ribosomal protein L14p (L23e) | 9.61E-86 | 0 | 98.36 | 100 |
| 104628.16_00510 | 50S ribosomal protein L24 | BN341_7410 | LSU ribosomal protein L24p (L26e) | 2.86E-41 | 0 | 87.67 | 94.52 |
| 104628.16_00513 | 30S ribosomal protein S8 | BN341_7380 | SSU ribosomal protein S8p (S15Ae) | 2.48E-82 | 0 | 87.79 | 94.66 |
| 104628.16_00514 | 50S ribosomal protein L6 | BN341_7360 | LSU ribosomal protein L6p (L9e) | 2.01E-102 | 0 | 89.54 | 97.39 |
| 104628.16_00515 | 50S ribosomal protein L18 | BN341_7350 | LSU ribosomal protein L18p (L5e) | 5.68E-62 | 0 | 82.20 | 88.98 |
| 104628.16_00529 | putative FAD-linked oxidoreductase | BN341_16590 | (S)-2-hydroxy-acid oxidase | 0 | 0 | 87.80 | 93.03 |
| 104628.16_00651 | heat shock protein GrpE | BN341_6100 | Heat shock protein GrpE | 1.11E-90 | 0 | 83.11 | 93.92 |
| 104628.16_00747 | Elongation factor G | BN341_5760 | Translation elongation factor G | 0 | 0 | 95.65 | 98.41 |
| 104628.16_00749 | Alcohol dehydrogenase | BN341_10660 | Alcohol dehydrogenase | 9.23E-41 | 26 | 32.65 | 48.30 |
| 104628.16_00896 | ATP synthase gamma chain | BN341_19770 | ATP synthase gamma chain | 0 | 2 | 84.11 | 92.05 |
| 104628.16_00961 | Phosphomethylpyrimidine synthase | BN341_15750 | Hydroxymethylpyrimidine phosphate synthase ThiC | 0 | 1 | 91.16 | 95.92 |
| 104628.16_00967 | Ubiquinol-cytochrome c reductase iron-sulfur subunit | BN341_7050 | Ubiquinol-cytochrome C reductase iron-sulfur subunit | 5.52E-81 | 0 | 82.93 | 91.46 |
| 104628.16_01071 | NifU-like protein | BN341_14600 | Iron-sulfur cluster assembly scaffold protein IscU/NifU-like | 0 | 0 | 93.25 | 97.55 |
| 104628.16_01111 | General stress protein 16U | BN341_2960 | Tellurium resistance protein TerD | 6.9E-120 | 0 | 85.42 | 92.71 |
| 104628.16_01123 | Thioredoxin reductase | BN341_12370 | Thioredoxin reductase | 1.05E-63 | 0 | 82.14 | 93.75 |
| 104628.16_01385 | hypothetical protein | BN341_15190 | hypothetical protein | 9.64E-65 | 0 | 52.75 | 72.53 |
| 104628.16_01444 | hypothetical protein | BN341_450 | hypothetical protein | 1.22E-34 | 28 | 42.86 | 58.86 |
| 104628.16_01454 | Chaperone protein ClpB | BN341_340 | ClpB protein | 0 | 30 | 79.14 | 87.76 |
| 104628.16_01554 | N-carbamoyl-D-amino acid hydrolase | BN341_5010 | N-carbamoylputrescine amidase (3.5.1.53) | 7.83E-154 | 6 | 68.77 | 80.73 |
| 104628.16_01600 | Phospho-2-dehydro-3-deoxyheptonate aldolase | BN341_13600 | 2-keto-3-deoxy-D-arabino-heptulosonate-7-phosphate synthase II | 0 | 2 | 81.72 | 87.81 |
| 104628.16_00290 | Shikimate kinase |  |  |  |  |  |  |
| 104628.16_00440 | 60 kDa chaperonin 1 |  |  |  |  |  |  |
| 104628.16_00726 | hypothetical protein |  |  |  |  |  |  |
| 104628.16_00911 | hypothetical protein |  |  |  |  |  |  |
| 104628.16_00960 | hypothetical protein |  |  |  |  |  |  |
| 104628.16_01016 | Cell wall-associated hydrolase |  |  |  |  |  |  |
