# Supplementary material for: Distinct transcriptome signatures of Helicobacter suis and Helicobacter heilmannii strains upon adherence to human gastric epithelial cells
Source: Vet Res. 2020 May 7;51:62. doi: 10.1186/s13567-020-00786-w (PMC7206758; doi:10.1186/s13567-020-00786-w)
Supplement: Supplementary file 9 — Additional file 9. H. heilmannii up-regulated genes with (47) and without (13) H. suis homologs according to BLASTp. [file 13567_2020_786_MOESM9_ESM.docx]

| ***H. heilmannii* genID** | **Description** | ***H. suis* genID** | **Description** | **E-value** | **Gaps** | **% identity** | **% positive match** |
| --- | --- | --- | --- | --- | --- | --- | --- |
| BN341_10170 | conserved hypothetical protein with DUF394 domain | 104628.16_00286 | hypothetical protein | 0 | 17 | 59.03 | 73.95 |
| BN341_10490 | Replicative DNA helicase | 104628.16_01215 | Replicative DNA helicase | 0 | 13 | 62.14 | 74.90 |
| BN341_1080 | Ferrous iron transport protein B | 104628.16_00363 | Ferrous iron transport protein B | 0 | 11 | 67.11 | 78.44 |
| BN341_11930 | Seryl-tRNA synthetase | 104628.16_01093 | Serine--tRNA ligase | 0 | 1 | 78.76 | 87.11 |
| BN341_11980 | secreted protein involved in flagellar motility | 104628.16_01088 | META domain protein | 7.04E-100 | 6 | 61.67 | 75.77 |
| BN341_120 | hypothetical protein | 104628.16_00009 | Helicobacter outer membrane protein | 4.2E-81 | 3 | 59.38 | 74.48 |
| BN341_130 | Acriflavin resistance protein / Multidrug efflux system CmeDEF | 104628.16_00010 | Multidrug resistance protein MdtC | 1.39E-34 | 0 | 82.35 | 95.59 |
| BN341_13030 | putative THIOREDOXIN | 104628.16_01254 | Thioredoxin | 3.94E-52 | 0 | 63.64 | 78.18 |
| BN341_14020 | conserved hypothetical protein | 104628.16_00932 | R.Pab1 restriction endonuclease | 7.6E-70 | 7 | 56.06 | 70.20 |
| BN341_14190 | Dihydrofolate synthase | 104628.16_00864 | Bifunctional protein FolC | 3.22E-139 | 8 | 57.48 | 72.44 |
| BN341_14880 | hypothetical protein | 104628.16_00120 | Malate-2H(+)/Na(+)-lactate antiporter | 0 | 4 | 76.67 | 86.00 |
| BN341_15110 | ATP-dependent Clp protease ATP-binding subunit ClpX | 104628.16_00205 | ATP-dependent Clp protease ATP-binding subunit ClpX | 0 | 2 | 86.87 | 93.78 |
| BN341_15280 | Transcription antitermination protein NusG | 104628.16_00220 | hypothetical protein | 4.91E-120 | 0 | 90.86 | 97.71 |
| BN341_160 | Probable outer membrane component of multidrug efflux pump | 104628.16_00012 | Outer membrane efflux protein | 0 | 2 | 76.77 | 90.04 |
| BN341_1640 | disulphide isomerase | 104628.16_01132 | hypothetical protein | 3.78E-120 | 3 | 66.53 | 80.65 |
| BN341_16620 | Membrane proteins related to metalloendopeptidases | 104628.16_00532 | Murein DD-endopeptidase MepM | 0 | 1 | 76.72 | 87.30 |
| BN341_16760 | Acetophenone carboxylase subunit Apc3 | 104628.16_00423 | Acetophenone carboxylase alpha subunit | 6.39E-18 | 2 | 33.08 | 57.69 |
| BN341_16830 | Flagellar assembly factor FliW | 104628.16_00416 | Flagellar assembly factor FliW 2 | 2.26E-77 | 0 | 85.08 | 94.03 |
| BN341_16960 | RNA polymerase sigma factor RpoD | 104628.16_00407 | RNA polymerase sigma factor RpoD | 0 | 5 | 87.07 | 94.48 |
| BN341_17040 | Urease beta subunit | 104628.16_00398 | Urease subunit beta | 0 | 0 | 95.78 | 98.24 |
| BN341_17100 | Urease accessory protein UreF | 104628.16_00395 | Urease accessory protein UreF | 1.58E-137 | 0 | 80.09 | 88.74 |
| BN341_19530 | Transcription termination factor Rho | 104628.16_00007 | hypothetical protein | 0 | 0 | 96.05 | 98.60 |
| BN341_19560 | Ferrochelatase, protoheme ferro-lyase | 104628.16_00004 | Ferrochelatase | 8.1E-166 | 0 | 70.42 | 84.24 |
| BN341_19710 | Methionyl-tRNA formyltransferase | 104628.16_00890 | Methionyl-tRNA formyltransferase | 1.28E-152 | 0 | 68.69 | 81.82 |
| BN341_19730 | ATP synthase B' chain | 104628.16_00892 | ATP synthase subunit b, sodium ion specific | 6.48E-55 | 0 | 64.54 | 86.52 |
| ***H. heilmannii* genID** | **Description** | ***H. suis* genID** | **Description** | **E-value** | **Gaps** | **% identity** | **% positive match** |
| BN341_19750 | ATP synthase delta chain | 104628.16_00894 | F0F1 ATP synthase subunit delta | 3.09E-80 | 0 | 63.64 | 79.55 |
| BN341_4150 | Biopolymer transport protein ExbD/TolR | 104628.16_00673 | Biopolymer transport protein ExbD | 1.13E-66 | 0 | 86.82 | 95.35 |
| BN341_4210 | FIG00710144: hypothetical protein | 104628.16_01350 | hypothetical protein | 1.67E-39 | 71 | 26.85 | 43.85 |
| BN341_4310 | Gamma-glutamyltranspeptidase | 104628.16_00820 | Gamma-glutamyltranspeptidase precursor | 0 | 0 | 82.97 | 92.03 |
| BN341_4620 | Molybdenum cofactor biosynthesis protein MoaA | 104628.16_01155 | Cyclic pyranopterin monophosphate synthase | 0 | 0 | 82.61 | 90.06 |
| BN341_470 | Putative transmembrane transport protein | 104628.16_01413 | Inner membrane transport protein YdhP | 0 | 1 | 68.83 | 82.08 |
| BN341_5220 | Beta-1,4-galactosyltransferase | 104628.16_00731 | Glycosyltransferase family 25 (LPS biosynthesis protein) | 6.52E-77 | 6 | 48.86 | 63.36 |
| BN341_5230 | Beta-1,4-galactosyltransferase | 104628.16_00731 | Glycosyltransferase family 25 (LPS biosynthesis protein) | 1.63E-68 | 6 | 47.35 | 62.45 |
| BN341_5540 | hypothetical protein | 104628.16_01566 | Response regulator ArlR | 5.71E-19 | 1 | 78.57 | 88.10 |
| BN341_6220 | UDP-N-acetylglucosamine 4,6-dehydratase | 104628.16_01117 | UDP-N-acetylglucosamine 4,6-dehydratase (inverting) | 0 | 0 | 82.26 | 89.91 |
| BN341_6230 | Phosphopantothenoylcysteine decarboxylase | 104628.16_01116 | Coenzyme A biosynthesis bifunctional protein CoaBC | 0 | 4 | 64.37 | 77.15 |
| BN341_6880 | A/G-specific adenine glycosylase | 104628.16_00732 | A/G-specific adenine glycosylase | 6.45E-154 | 1 | 66.36 | 78.50 |
| BN341_7530 | SSU ribosomal protein S10p (S20e) | 104628.16_00498 | 30S ribosomal protein S10 | 5.51E-71 | 0 | 97.12 | 99.04 |
| BN341_8050 | Carbon starvation protein A | 104628.16_00734 | Carbon starvation protein A | 0 | 0 | 78.95 | 88.84 |
| BN341_8270 | hypothetical protein | 104628.16_01286 | DNA gyrase subunit A | 0.009 | 1 | 32.65 | 61.22 |
| BN341_8630 | hypothetical protein | 104628.16_01588 | hypothetical protein | 2.9E-99 | 7 | 59.92 | 70.24 |
| BN341_8650 | hypothetical protein | 104628.16_01588 | hypothetical protein | 2.9E-99 | 7 | 59.92 | 70.24 |
| BN341_8660 | hypothetical protein | 104628.16_01587 | hypothetical protein | 3.41E-54 | 4 | 47.18 | 66.67 |
| BN341_9640 | Foldase protein PrsA precursor | 104628.16_00389 | Putative peptidyl-prolyl *cis,trans*-isomerase Cbf2 precursor | 7.53E-175 | 2 | 78.19 | 89.26 |
| BN341_9650 | Fructose-bisphosphate aldolase class II | 104628.16_00390 | Fructose-bisphosphate aldolase | 0 | 0 | 85.99 | 93.81 |
| BN341_9870 | hypothetical protein | 104628.16_01622 | putative transposase | 5.14E-90 | 2 | 68.59 | 81.15 |
| BN341_9900 | hypothetical protein | 104628.16_01622 | putative transposase | 5.14E-90 | 2 | 68.59 | 81.15 |
| BN341_11390 | hypothetical protein |  |  |  |  |  |  |
| BN341_12170 | DNA-cytosine methyltransferase |  |  |  |  |  |  |
| BN341_140 | hypothetical protein |  |  |  |  |  |  |
| BN341_14920 | hypothetical protein |  |  |  |  |  |  |
| BN341_14970 | hypothetical protein |  |  |  |  |  |  |
| ***H. heilmannii* genID** | **Description** | ***H. suis* genID** | **Description** | **E-value** | **Gaps** | **% identity** | **% positive match** |
| BN341_18980 | hypothetical protein |  |  |  |  |  |  |
| BN341_19320 | Homolog of fucose/glucose/galactose permeases |  |  |  |  |  |  |
| BN341_3880 | hypothetical protein |  |  |  |  |  |  |
| BN341_4250 | SSU ribosomal protein S9p (S16e) |  |  |  |  |  |  |
| BN341_4260 | LSU ribosomal protein L13p (L13Ae) |  |  |  |  |  |  |
| BN341_5080 | hypothetical protein |  |  |  |  |  |  |
| BN341_5240 | Putative membrane protein YeiH |  |  |  |  |  |  |
| BN341_6170 | Iron(III) dicitrate transport protein FecA |  |  |  |  |  |  |
