# Supplementary material for: Distinct transcriptome signatures of Helicobacter suis and Helicobacter heilmannii strains upon adherence to human gastric epithelial cells
Source: Vet Res. 2020 May 7;51:62. doi: 10.1186/s13567-020-00786-w (PMC7206758; doi:10.1186/s13567-020-00786-w)
Supplement: Supplementary file 10 — Additional file 10. H. heilmannii down-regulated genes with (74) and without (9) H. suis homologs according to BLASTp. [file 13567_2020_786_MOESM10_ESM.docx]

| ***H. heilmannii* genID** | **Description** | ***H. suis* genID** | **Description** | **E-value** | **Gaps** | **% identity** | **% positive match** |
| --- | --- | --- | --- | --- | --- | --- | --- |
| BN341_10030 | dicarboxylic acid transporter PcaT | 104628.16_00158 | Alpha-ketoglutarate permease | 0 | 0 | 78.55 | 90.36 |
| BN341_10590 | hypothetical protein | 104628.16_00707 | Putative F0F1-ATPase subunit (ATPase_gene1) | 6.54E-46 | 0 | 78.16 | 86.21 |
| BN341_11500 | RNA-binding protein | 104628.16_01182 | RNA recognition motif. (a.k.a. RRM, RBD, or RNP domain) | 1.28E-32 | 0 | 94.12 | 98.04 |
| BN341_12060 | KH domain RNA binding protein YlqC | 104628.16_00458 | hypothetical protein | 9.66E-42 | 1 | 67.78 | 84.44 |
| BN341_12070 | SSU ribosomal protein S16p | 104628.16_00459 | 30S ribosomal protein S16 | 8.27E-47 | 0 | 87.84 | 95.95 |
| BN341_12340 | hypothetical protein | 104628.16_00858 | Bacterial protein of unknown function (YtfJ_HI0045) | 6.02E-99 | 0 | 81.66 | 87.57 |
| BN341_12690 | Polyferredoxin NapH (periplasmic nitrate reductase) | 104628.16_00878 | Putative electron transport protein YccM | 3.49E-143 | 2 | 75.67 | 88.97 |
| BN341_12930 | Cytochrome C553 (soluble cytochrome f) | 104628.16_01264 | Cytochrome c-553 precursor | 1.02E-52 | 0 | 72.00 | 85.00 |
| BN341_13080 | hypothetical protein | 104628.16_01147 | hypothetical protein | 1.42E-25 | 27 | 31.14 | 49.12 |
| BN341_13120 | hypothetical protein | 104628.16_01147 | hypothetical protein | 1.42E-25 | 27 | 31.14 | 49.12 |
| BN341_13230 | Acyl-phosphate:glycerol-3-phosphate O-acyltransferase PlsY | 104628.16_00093 | Glycerol-3-phosphate acyltransferase | 2.03E-118 | 1 | 74.55 | 88.18 |
| BN341_13680 | CDP-diacylglycerol--serine O-phosphatidyltransferase | 104628.16_00487 | CDP-alcohol phosphatidyltransferase | 6.82E-123 | 0 | 76.62 | 87.45 |
| BN341_1560 | Outer membrane protein | 104628.16_01138 | Putative outer membrane protein | 0 | 1 | 71.68 | 85.18 |
| BN341_15670 | GTP-binding and nucleic acid-binding protein YchF | 104628.16_00952 | Ribosome-binding ATPase YchF | 0 | 1 | 81.97 | 90.16 |
| BN341_16270 | Phosphoserine phosphatase | 104628.16_00594 | Phosphoserine phosphatase | 1.02E-94 | 0 | 65.52 | 76.85 |
| BN341_16320 | Aspartyl-tRNA(Asn) amidotransferase subunit C | 104628.16_00272 | Glutamyl-tRNA(Gln) amidotransferase subunit C | 2.38E-31 | 0 | 54.26 | 73.40 |
| BN341_16400 | Hydrogenase maturation protease | 104628.16_00077 | hydrogenase 2 maturation endopeptidase | 2.78E-74 | 2 | 68.15 | 83.44 |
| BN341_16430 | Quinone-reactive Ni/Fe-hydrogenase small chain precursor | 104628.16_00073 | Quinone-reactive Ni/Fe-hydrogenase small chain precursor | 0 | 0 | 82.63 | 91.05 |
| BN341_1710 | hypothetical protein | 104628.16_01302 | hypothetical protein | 1.05E-46 | 0 | 86.08 | 96.20 |
| BN341_17150 | putative type II DNA modification enzyme (methyltranferase) | 104628.16_01217 | DNA adenine methyltransferase YhdJ | 0.0000671 | 13 | 25.77 | 46.39 |
| BN341_17500 | Prolipoprotein diacylglyceryl transferase | 104628.16_00760 | Prolipoprotein diacylglyceryl transferase | 3.06E-159 | 0 | 80.15 | 90.81 |
| BN341_17990 | 6,7-dimethyl-8-ribityllumazine synthase | 104628.16_00346 | 6,7-dimethyl-8-ribityllumazine synthase | 1.77E-88 | 0 | 76.82 | 92.72 |
| BN341_180 | DNA-binding protein HU | 104628.16_00014 | DNA-binding protein HU | 3.03E-54 | 0 | 88.89 | 95.56 |
| BN341_18000 | Transcription termination protein NusB | 104628.16_00347 | hypothetical protein | 8.49E-65 | 0 | 67.94 | 80.92 |
| BN341_18090 | RecA protein | 104628.16_00356 | recombinase A | 0 | 1 | 87.65 | 93.82 |
| ***H. heilmannii* genID** | **Description** | ***H. suis* genID** | **Description** | **E-value** | **Gaps** | **% identity** | **% positive match** |
| BN341_18110 | hypothetical protein | 104628.16_00358 | hypothetical protein | 2.36E-87 | 3 | 62.84 | 79.78 |
| BN341_18190 | 3-methyl-2-oxobutanoate hydroxymethyltransferase | 104628.16_01054 | 3-methyl-2-oxobutanoate hydroxymethyltransferase | 2.95E-83 | 0 | 75.16 | 88.89 |
| BN341_18660 | hypothetical protein | 104628.16_00980 | Cupin domain protein | 1.13E-55 | 0 | 68.81 | 84.40 |
| BN341_18910 | Holo-[acyl-carrier protein] synthase | 104628.16_00051 | Holo-[acyl-carrier-protein] synthase | 1.19E-49 | 0 | 59.83 | 78.63 |
| BN341_2070 | NADH dehydrogenase | 104628.16_00797 | NADH dehydrogenase-like protein | 6.25E-84 | 4 | 59.14 | 73.56 |
| BN341_2080 | Threonine dehydrogenase and related Zn-dependent dehydrogenases | 104628.16_00796 | NADP-dependent isopropanol dehydrogenase | 0 | 0 | 83.61 | 92.90 |
| BN341_220 | hypothetical protein | 104628.16_00018 | hypothetical protein | 2.65E-24 | 2 | 56.32 | 65.52 |
| BN341_250 | hypothetical protein | 104628.16_00018 | hypothetical protein | 2.65E-24 | 2 | 56.32 | 65.52 |
| BN341_260 | hypothetical protein | 104628.16_00018 | hypothetical protein | 5.38E-20 | 1 | 57.14 | 70.00 |
| BN341_2750 | hypothetical protein | 104628.16_01017 | hypothetical protein | 7.45E-20 | 6 | 53.85 | 65.38 |
| BN341_2960 | Tellurium resistance protein TerD | 104628.16_01111 | General stress protein 16U | 6.73E-120 | 0 | 85.42 | 92.71 |
| BN341_2970 | Tellurium resistance protein | 104628.16_01112 | Stress response protein SCP2 | 5.63E-105 | 1 | 81.91 | 89.95 |
| BN341_3090 | outer membrane protein 13 | 104628.16_01239 | Helicobacter outer membrane protein | 3.84E-130 | 0 | 74.68 | 83.54 |
| BN341_3170 | 8-amino-7-oxononanoate synthase | 104628.16_01247 | 8-amino-7-oxononanoate synthase | 1.78E-180 | 0 | 67.69 | 81.06 |
| BN341_3310 | hypothetical protein | 104628.16_00046 | Helicobacter outer membrane protein | 1.1E-106 | 0 | 81.25 | 89.77 |
| BN341_3320 | hypothetical protein | 104628.16_00045 | hypothetical protein | 2.97E-50 | 15 | 59.54 | 74.81 |
| BN341_3450 | outer membrane protein (omp30) | 104628.16_00036 | Helicobacter outer membrane protein | 1.39E-115 | 18 | 61.94 | 76.12 |
| BN341_4040 | C4-dicarboxylate transporter DcuA | 104628.16_01034 | Anaerobic C4-dicarboxylate transporter DcuA | 9.42E-142 | 2 | 71.63 | 82.62 |
| BN341_4060 | putative | 104628.16_01032 | hypothetical protein | 2.64E-77 | 1 | 63.35 | 78.26 |
| BN341_410 | hypothetical protein | 104628.16_01448 | hypothetical protein | 1.48E-93 | 0 | 81.70 | 94.12 |
| BN341_4170 | Integral membrane protein | 104628.16_00333 | Modulator of FtsH protease YccA | 1.11E-144 | 2 | 86.03 | 95.20 |
| BN341_4450 | 2-oxoglutarate oxidoreductase, delta subunit, putative | 104628.16_01341 | 2-oxoglutarate-acceptor oxidoreductase subunit OorD | 4.75E-69 | 0 | 84.96 | 92.92 |
| BN341_4480 | hypothetical protein | 104628.16_01338 | FeoA domain protein | 5.87E-32 | 0 | 65.33 | 76.00 |
| BN341_4570 | [NiFe] hydrogenase metallocenter assembly protein HypD | 104628.16_01150 | Hydrogenase expression/formation protein HypD | 0 | 0 | 72.95 | 84.70 |
| BN341_4810 | hypothetical protein | 104628.16_00466 | hypothetical protein | 2.47E-25 | 0 | 67.86 | 83.93 |
| BN341_4820 | Formate dehydrogenase, major subunit | 104628.16_00467 | Putative formate dehydrogenase | 2.73E-112 | 0 | 94.25 | 97.13 |
| BN341_4940 | putative | 104628.16_01559 | lipid A 1-phosphatase | 1.42E-118 | 3 | 82.74 | 87.31 |
| ***H. heilmannii* genID** | **Description** | ***H. suis* genID** | **Description** | **E-value** | **Gaps** | **% identity** | **% positive match** |
| BN341_5040 | hypothetical protein | 104628.16_00472 | Multidrug export protein MepA | 0 | 1 | 77.16 | 87.02 |
| BN341_510 | outer membrane protein (omp4) | 104628.16_00212 | Helicobacter outer membrane protein | 9.65E-48 | 12 | 43.98 | 57.59 |
| BN341_5350 | hypothetical protein | 104628.16_00435 | hypothetical protein | 9.24E-65 | 0 | 75.22 | 88.50 |
| BN341_5740 | SSU ribosomal protein S12p (S23e) | 104628.16_00745 | 30S ribosomal protein S12 | 5.63E-90 | 3 | 94.20 | 94.93 |
| BN341_5900 | hypothetical protein | 104628.16_01017 | hypothetical protein | 7.45E-20 | 6 | 53.85 | 65.38 |
| BN341_5960 | hypothetical protein | 104628.16_00257 | hypothetical protein | 0.000719 | 4 | 42.22 | 55.56 |
| BN341_6100 | Heat shock protein GrpE | 104628.16_00651 | heat shock protein GrpE | 1.72E-89 | 0 | 84.03 | 94.44 |
| BN341_6140 | UDP-N-acetylglucosamine--N-acetylmuramyl-(pentapeptide) pyrophosphoryl-undecaprenol N-acetylglucosamine transferase | 104628.16_00647 | UDP-N-acetylglucosamine--N-acetylmuramyl-(pentapeptide) pyrophosphoryl-undecaprenol N-acetylglucosamine transferase | 0 | 0 | 71.83 | 85.92 |
| BN341_6150 | Flagellar assembly factor FliW | 104628.16_00646 | Flagellar assembly factor FliW 1 | 7.96E-87 | 1 | 88.15 | 95.56 |
| BN341_6370 | hypothetical protein | 104628.16_01273 | hypothetical protein | 3.06E-42 | 10 | 55.07 | 71.01 |
| BN341_6380 | outer membrane protein 27 | 104628.16_01274 | Helicobacter outer membrane protein | 1.27E-91 | 6 | 66.82 | 78.34 |
| BN341_6440 | putative protease (EC:3.4.-) | 104628.16_01173 | putative protease YhbU precursor | 4.01E-58 | 0 | 70.34 | 88.14 |
| BN341_6740 | Molybdenum cofactor biosynthesis protein MoaC | 104628.16_00321 | Cyclic pyranopterin monophosphate synthase accessory protein | 6.24E-77 | 0 | 71.43 | 81.17 |
| BN341_6750 | Molybdopterin biosynthesis Mog protein, molybdochelatase | 104628.16_00322 | Molybdopterin adenylyltransferase | 3.9E-105 | 2 | 83.52 | 89.20 |
| BN341_7680 | hypothetical protein | 104628.16_01384 | Putative beta-lactamase HcpC precursor | 6.69E-21 | 0 | 43.27 | 62.50 |
| BN341_890 | TrkA | 104628.16_00095 | Ktr system potassium uptake protein A | 1.58E-115 | 2 | 72.48 | 87.16 |
| BN341_9340 | Peptidyl-prolyl *cis,trans*-isomerase | 104628.16_01431 | Peptidyl-prolyl *cis,trans*-isomerase B | 2.15E-96 | 0 | 79.50 | 86.96 |
| BN341_9350 | Carbon storage regulator | 104628.16_01430 | hypothetical protein | 2.53E-40 | 0 | 78.21 | 93.59 |
| BN341_9360 | 4-diphosphocytidyl-2-C-methyl-D-erythritol kinase | 104628.16_01429 | 4-diphosphocytidyl-2-C-methyl-D-erythritol kinase | 1E-77 | 5 | 54.69 | 68.75 |
| BN341_9370 | tmRNA-binding protein SmpB | 104628.16_01428 | SsrA-binding protein | 3.47E-85 | 0 | 79.73 | 89.86 |
| BN341_9620 | Protein crcB homolog | 104628.16_00387 | Putative fluoride ion transporter CrcB | 1.09E-57 | 0 | 81.03 | 87.93 |
| BN341_9940 | hypothetical protein | 104628.16_01615 | hypothetical protein | 1.16E-44 | 0 | 57.80 | 76.15 |
| BN341_11170 | hypothetical protein |  |  |  |  |  |  |
| BN341_14810 | hypothetical protein |  |  |  |  |  |  |
| BN341_16510 | hypothetical protein |  |  |  |  |  |  |
| BN341_17260 | Mn2+/Fe2+ transporter, NRAMP family |  |  |  |  |  |  |
| ***H. heilmannii* genID** | **Description** | ***H. suis* genID** | **Description** | **E-value** | **Gaps** | **% identity** | **% positive match** |
| BN341_18480 | unknown |  |  |  |  |  |  |
| BN341_4220 | hypothetical protein |  |  |  |  |  |  |
| BN341_4690 | hypothetical protein (EC:3.1.21.4) |  |  |  |  |  |  |
| BN341_6910 | Modification methylase |  |  |  |  |  |  |
| BN341_7960 | hypothetical protein |  |  |  |  |  |  |
| BN341_11170 | hypothetical protein |  |  |  |  |  |  |
