# Supplementary material for: Distinct transcriptome signatures of Helicobacter suis and Helicobacter heilmannii strains upon adherence to human gastric epithelial cells
Source: Vet Res. 2020 May 7;51:62. doi: 10.1186/s13567-020-00786-w (PMC7206758; doi:10.1186/s13567-020-00786-w)
Supplement: Supplementary file 11 — Additional file 11. Classification of up-regulated H. suis genes in cases compared to controls according to their function. [file 13567_2020_786_MOESM11_ESM.docx]

| **Functional class** | **Gene** | **Description** |
| --- | --- | --- |
| DNA modification/binding | 104628.16_01410 | DNA methylase |
|  | 104628.16_00438 | DNA primase |
|  | 104628.16_00174 | tumor necrosis factor alpha-inducing protein |
| Transcription | 104628.16_00759 | Putative nickel-responsive regulator |
| Translation | 104628.16_01377 | 30S ribosomal protein S20 |
| (Transmembrane) transport | 104628.16_00173 | Mechanosensitive ion channel |
|  | 104628.16_01165 | putative copper-transporting ATPase PacS |
|  | 104628.16_00302 | Oligopeptide-binding protein AppA precursor |
|  | 104628.16_00868 | preprotein translocase subunit SecF |
|  | 104628.16_00303 | Nickel transport system permease protein NikB |
|  | 104628.16_01022 | ABC-2 family transporter protein |
|  | 104628.16_01571 | putative phospholipid ABC transporter permease protein MlaE |
| Oxidation-reduction | 104628.16_01497 | NAD(P)H-quinone oxidoreductase subunit 3 |
|  | 104628.16_01489 | NADH-quinone oxidoreductase subunit J |
|  | 104628.16_01486 | NAD(P)H-quinone oxidoreductase chain 4 1 |
|  | 104628.16_00482 | putative oxidoreductase YdgJ |
|  | 104628.16_00155 | Fumarate reductase cytochrome b subunit |
| Biosynthetic process | 104628.16_01539 | CDP-diacylglycerol--glycerol-3-phosphate 3-phosphatidyltransferase |
|  | 104628.16_01099 | Thymidylate kinase |
|  | 104628.16_01404 | putative inorganic polyphosphate/ATP-NAD kinase |
|  | 104628.16_01189 | Octaprenyl-diphosphate synthase |
|  | 104628.16_00145 | 3-phosphoshikimate 1-carboxyvinyltransferase |
|  | 104628.16_00004 | Ferrochelatase |
|  | 104628.16_00789 | Farnesyl diphosphate synthase |
|  | 104628.16_00761 | Ribosomal large subunit pseudouridine synthase D |
|  | 104628.16_00450 | Ribosomal protein S12 methylthiotransferase RimO |
|  | 104628.16_01557 | L-seryl-tRNA(Sec) selenium transferase |
| Metabolic process | 104628.16_01297 | Phosphorylase superfamily protein |
|  | 104628.16_00395 | Urease accessory protein UreF |
|  | 104628.16_01201 | Putative phosphoribosyl transferase/MT0597 |
|  | 104628.16_01030 | Transaldolase |
|  | 104628.16_01363 | Glycosyltransferase family 9 (heptosyltransferase) |
|  | 104628.16_00843 | Amidophosphoribosyltransferase precursor |
|  | 104628.16_01324 | D-glycero-alpha-D-manno-heptose-1,7-bisphosphate 7-phosphatase |
|  | 104628.16_00393 | Urease accessory protein UreH |
| Chemotaxis and motility | 104628.16_01371 | Methyl-accepting chemotaxis protein 4 |
|  | 104628.16_01061 | Methyl-accepting chemotaxis protein PctC |
|  | 104628.16_01096 | flagellar basal body P-ring biosynthesis protein FlgA |
| Unknown | 104628.16_01441 | Helix-turn-helix domain protein |
|  | 104628.16_01010 | Acyl-CoA thioester hydrolase YbgC |
|  | 104628.16_01569 | paraquat-inducible protein B |
|  | 104628.16_01546 | Fic/DOC family protein |
|  | 104628.16_01370 | CobQ/CobB/MinD/ParA nucleotide binding domain protein |
|  | 104628.16_01560 | putative undecaprenyl-phosphate N-acetylglucosaminyl 1-phosphate transferase |
|  | 104628.16_01166 | tRNA1(Val) (adenine(37)-N6)-methyltransferase |
|  | 104628.16_01253 | 3-deoxy-manno-octulosonate cytidylyltransferase |
|  | 104628.16_01310 | Soluble lytic murein transglycosylase precursor |
|  | 104628.16_00764 | GTP cyclohydrolase 1 type 2 |
|  | 104628.16_01120 | PD-(D/E)XK nuclease superfamily protein |
|  | 104628.16_00348 | ribonuclease BN/unknown domain fusion protein |
