# Supplementary material for: Distinct transcriptome signatures of Helicobacter suis and Helicobacter heilmannii strains upon adherence to human gastric epithelial cells
Source: Vet Res. 2020 May 7;51:62. doi: 10.1186/s13567-020-00786-w (PMC7206758; doi:10.1186/s13567-020-00786-w)
Supplement: Supplementary file 12 — Additional file 12. Classification of up-regulated H. heilmannii genes in cases compared to controls according to their function. [file 13567_2020_786_MOESM12_ESM.docx]

| **Functional class** | **Gene** | **Description** |
| --- | --- | --- |
| DNA repair | BN341_6880 | A/G-specific adenine glycosylase |
| DNA replication | BN341_10490 | Replicative DNA helicase |
| Transcription | BN341_16960 | RNA polymerase sigma factor RpoD |
|  | BN341_19530 | Transcription termination factor Rho |
|  | BN341_15280 | Transcription antitermination protein NusG |
| Translation | BN341_11930 | Seryl-tRNA synthetase |
|  | BN341_4260 | LSU ribosomal protein L13p (L13Ae) |
|  | BN341_7530 | SSU ribosomal protein S10p (S20e) |
|  | BN341_4250 | SSU ribosomal protein S9p (S16e) |
| (Transmembrane) transport | BN341_130 | Acriflavin resistance protein / Multidrug efflux system CmeDEF |
|  | BN341_1080 | Ferrous iron transport protein B |
|  | BN341_19730 | ATP synthase B' chain |
|  | BN341_470 | Putative transmembrane transport protein |
|  | BN341_4150 | Biopolymer transport protein ExbD/TolR |
|  | BN341_160 | Probable outer membrane component of multidrug efflux pump |
|  | BN341_19750 | ATP synthase delta chain |
| Biosynthetic process | BN341_19560 | Ferrochelatase, protoheme ferro-lyase |
|  | BN341_19710 | Methionyl-tRNA formyltransferase |
|  | BN341_4620 | Molybdenum cofactor biosynthesis protein MoaA |
|  | BN341_14190 | Dihydrofolate synthase |
|  | BN341_6230 | Phosphopantothenoylcysteine decarboxylase |
| Metabolic process | BN341_17100 | Urease accessory protein UreF |
|  | BN341_17040 | Urease beta subunit |
|  | BN341_9650 | Fructose-bisphosphate aldolase class II |
| Catabolic process | BN341_4310 | Gamma-glutamyltranspeptidase |
| Motility | BN341_16830 | Flagellar assembly factor FliW |
|  | BN341_11980 | secreted protein involved in flagellar motility |
| Protein folding | BN341_15110 | ATP-dependent Clp protease ATP-binding subunit ClpX |
| Response to starvation | BN341_8050 | Carbon starvation protein A |
| Unknown | BN341_5230 | Beta-1,4-galactosyltransferase |
|  | BN341_5220 | Beta-1,4-galactosyltransferase |
|  | BN341_6170 | Iron(III) dicitrate transport protein FecA |
|  | BN341_9640 | Foldase protein PrsA precursor |
|  | BN341_16620 | Membrane proteins related to metalloendopeptidases |
|  | BN341_10170 | conserved hypothetical protein with DUF394 domain |
|  | BN341_12170 | DNA-cytosine methyltransferase |
|  | BN341_6220 | UDP-N-acetylglucosamine 4,6-dehydratase |
|  | BN341_13030 | putative THIOREDOXIN |
|  | BN341_19320 | Homolog of fucose/glucose/galactose permeases |
|  | BN341_5240 | Putative membrane protein YeiH |
|  | BN341_1640 | disulphide isomerase |
|  | BN341_16760 | Acetophenone carboxylase subunit Apc3 |
