# Supplementary material for: Distinct transcriptome signatures of Helicobacter suis and Helicobacter heilmannii strains upon adherence to human gastric epithelial cells
Source: Vet Res. 2020 May 7;51:62. doi: 10.1186/s13567-020-00786-w (PMC7206758; doi:10.1186/s13567-020-00786-w)
Supplement: Supplementary file 13 — Additional file 13. Classification of down-regulated H. suis genes in cases compared to controls according to their function. [file 13567_2020_786_MOESM13_ESM.docx]

| **Functional class** | **Gene** | **Description** |
| --- | --- | --- |
| DNA repair | 104628.16_00356 | recombinase A |
| DNA modification/binding | 104628.16_00081 | Modification methylase DpnIIB |
| Translation | 104628.16_00747 | Elongation factor G |
|  | 104628.16_00222 | 50S ribosomal protein L1 |
|  | 104628.16_00500 | 50S ribosomal protein L4 |
|  | 104628.16_00505 | 30S ribosomal protein S3 |
|  | 104628.16_00515 | 50S ribosomal protein L18 |
|  | 104628.16_00510 | 50S ribosomal protein L24 |
|  | 104628.16_00499 | 50S ribosomal protein L3 |
|  | 104628.16_00514 | 50S ribosomal protein L6 |
|  | 104628.16_00506 | 50S ribosomal protein L16 |
|  | 104628.16_00498 | 30S ribosomal protein S10 |
|  | 104628.16_00513 | 30S ribosomal protein S8 |
|  | 104628.16_00509 | 50S ribosomal protein L14 |
|  | 104628.16_00507 | 50S ribosomal protein L29 |
|  | 104628.16_00224 | 50S ribosomal protein L7/L12 |
| (Transmembrane) transport | 104628.16_00313 | Dipeptide transport system permease protein DppC |
|  | 104628.16_00896 | ATP synthase gamma chain |
| Oxidation-reduction | 104628.16_00154 | Fumarate reductase flavoprotein subunit |
|  | 104628.16_00529 | putative FAD-linked oxidoreductase |
|  | 104628.16_00967 | Ubiquinol-cytochrome c reductase iron-sulfur subunit |
|  | 104628.16_01123 | Thioredoxin reductase |
|  | 104628.16_00749 | Alcohol dehydrogenase |
| Biosynthetic process | 104628.16_00282 | Acyl carrier protein |
|  | 104628.16_01600 | Phospho-2-dehydro-3-deoxyheptonate aldolase |
|  | 104628.16_00283 | 3-oxoacyl-[acyl-carrier-protein] synthase 2 |
|  | 104628.16_00204 | Rod shape-determining protein MreB |
|  | 104628.16_00223 | 50S ribosomal protein L10 |
|  | 104628.16_00961 | Phosphomethylpyrimidine synthase |
|  | 104628.16_01071 | NifU-like protein |
| Metabolic process | 104628.16_00398 | Urease subunit beta |
|  | 104628.16_01554 | N-carbamoyl-D-amino acid hydrolase |
|  | 104628.16_00008 | Glutamate racemase |
|  | 104628.16_01454 | Chaperone protein ClpB |
| Protein folding | 104628.16_00651 | heat shock protein GrpE |
|  | 104628.16_00440 | 60 kDa chaperonin 1 |
| Response to stress | 104628.16_01111 | General stress protein 16U |
| Unknown | 104628.16_00269 | UDP-2-acetamido-3-amino-2,3-dideoxy-D-glucuronate N-acetyltransferase |
|  | 104628.16_00429 | Acetophenone carboxylase delta subunit |
|  | 104628.16_00290 | Shikimate kinase |
|  | 104628.16_01016 | Cell wall-associated hydrolase |
