# Supplementary material for: Distinct transcriptome signatures of Helicobacter suis and Helicobacter heilmannii strains upon adherence to human gastric epithelial cells
Source: Vet Res. 2020 May 7;51:62. doi: 10.1186/s13567-020-00786-w (PMC7206758; doi:10.1186/s13567-020-00786-w)
Supplement: Supplementary file 14 — Additional file 14. Classification of down-regulated H. heilmannii genes in cases compared to controls according to their function. [file 13567_2020_786_MOESM14_ESM.docx]

| **Functional class** | **Gene** | **Description** |
| --- | --- | --- |
| DNA repair | BN341_18090 | RecA protein |
| DNA modification/binding | BN341_17150 | Putative type II DNA modification enzyme (methyltransferase) |
|  | BN341_18480 | unknown |
|  | BN341_180 | DNA-binding protein HU |
| Transcription | BN341_18000 | Transcription termination protein NusB |
| Translation | BN341_16320 | Aspartyl-tRNA(Asn) amidotransferase subunit C |
|  | BN341_12070 | SSU ribosomal protein S16p |
|  | BN341_5740 | SSU ribosomal protein S12p (S23e) |
| (Transmembrane) transport | BN341_4040 | C4-dicarboxylate transporter DcuA |
|  | BN341_890 | TrkA |
|  | BN341_10030 | dicarboxylic acid transporter PcaT |
|  | BN341_17260 | Mn2+/Fe2+ transporter, NRAMP family |
| Cell envelope | BN341_3090 | outer membrane protein 13 (omp13) |
|  | BN341_4170 | Integral membrane protein |
|  | BN341_6380 | outer membrane protein 27 (omp27) |
|  | BN341_510 | outer membrane protein (omp4) |
|  | BN341_1560 | outer membrane protein |
|  | BN341_3450 | outer membrane protein (omp30) |
| Oxidation-reduction | BN341_2070 | NADH dehydrogenase |
|  | BN341_4820 | Formate dehydrogenase-O, major subunit |
|  | BN341_16430 | Quinone-reactive Ni/Fe-hydrogenase small chain precursor |
|  | BN341_2080 | Threonine dehydrogenase and related Zn-dependent dehydrogenases |
|  | BN341_4450 | 2-oxoglutarate oxidoreductase, delta subunit, putative |
| Biosynthetic process | BN341_6750 | Molybdopterin biosynthesis Mog protein, molybdochelatase |
|  | BN341_18190 | 3-methyl-2-oxobutanoate hydroxymethyltransferase |
|  | BN341_13680 | CDP-diacylglycerol--serine O-phosphatidyltransferase |
|  | BN341_3170 | 8-amino-7-oxononanoate synthase |
|  | BN341_13230 | Acyl-phosphate:glycerol-3-phosphate O-acyltransferase PlsY |
|  | BN341_17500 | Prolipoprotein diacylglyceryl transferase |
|  | BN341_18910 | Holo-[acyl-carrier protein] synthase |
|  | BN341_17990 | 6,7-dimethyl-8-ribityllumazine synthase |
|  | BN341_6740 | Molybdenum cofactor biosynthesis protein MoaC |
|  | BN341_9360 | 4-diphosphocytidyl-2-C-methyl-D-erythritol kinase |
| Metabolic process | BN341_6140 | UDP-N-acetylglucosamine-N-acetylmuramyl-(pentapeptide) pyrophosphoryl-undecaprenol N-acetylglucosamine transferase |
|  | BN341_9350 | Carbon storage regulator |
| Motility | BN341_6150 | Flagellar assembly factor FliW |
| Protein folding | BN341_6100 | Heat shock protein GrpE |
|  | BN341_9340 | Peptidyl-prolyl *cis,trans*-isomerase |
| Response to stress | BN341_2970 | Tellurium resistance protein |
|  | BN341_2960 | Tellurium resistance protein TerD |
| Unknown | BN341_4060 | putative |
|  | BN341_4940 | putative |
|  | BN341_12060 | KH domain RNA binding protein YlqC |
|  | BN341_6440 | putative protease(EC:3.4.-) |
|  | BN341_9620 | Protein crcB homolog |
|  | BN341_12690 | Polyferredoxin NapH (periplasmic nitrate reductase) |
|  | BN341_16270 | Phosphoserine phosphatase |
|  | BN341_11500 | RNA-binding protein |
|  | BN341_12930 | Cytochrome C553 (soluble cytochrome f) |
|  | BN341_4570 | [NiFe] hydrogenase metallocenter assembly protein HypD |
|  | BN341_15670 | GTP-binding and nucleic acid-binding protein YchF |
|  | BN341_9370 | tmRNA-binding protein SmpB |
|  | BN341_6910 | Modification methylase |
|  | BN341_16400 | Hydrogenase maturation protease |
